# Catalytic nitrogen fixation using visible light energy

**DOI:** 10.1038/s41467-022-34984-1

**Published:** 2022-12-01

**Authors:** Yuya Ashida, Yuto Onozuka, Kazuya Arashiba, Asuka Konomi, Hiromasa Tanaka, Shogo Kuriyama, Yasuomi Yamazaki, Kazunari Yoshizawa, Yoshiaki Nishibayashi

**Affiliations:** 1 Department of Applied Chemistry, School of Engineering, The University of Tokyo, Bunkyo-ku, Tokyo Japan; 2 Institute for Materials Chemistry and Engineering, Kyushu University, Nishi-ku, Fukuoka Japan; 3 School of Liberal Arts and Sciences, Daido University, Minami-ku, Nagoya Japan

**Keywords:** Organometallic chemistry, Homogeneous catalysis, Materials for energy and catalysis

## Abstract

The synthesis of ammonia from atmospheric dinitrogen, nitrogen fixation, is one of the essential reactions for human beings. Because the current industrial nitrogen fixation depends on dihydrogen produced from fossil fuels as raw material, the development of a nitrogen fixation reaction that relies on the energy provided by renewable energy, such as visible light, is an important research goal from the viewpoint of sustainable chemistry. Herein, we establish an iridium- and molybdenum-catalysed process for synthesizing ammonia from dinitrogen under ambient reaction conditions and visible light irradiation. In this reaction system, iridium complexes and molybdenum triiodide complexes bearing *N*-heterocyclic carbene-based pincer ligands act as cooperative catalysts to activate 9,10-dihydroacridine and dinitrogen, respectively. The reaction of dinitrogen with 9,10-dihydroacridine is not thermodynamically favoured, and it only takes place under visible light irradiation. Therefore, the described reaction system is one that affords visible light energy–driven ammonia formation from dinitrogen catalytically.

## Abstract (web summary)

The development of a nitrogen fixation process that relies on the renewable energy, such as visible light, is an important goal in sustainable chemistry. Here, authors establish an iridium and molybdenum-catalysed ammonia formation from dinitrogen driven by visible light under ambient reaction conditions.

## Introduction

**Fig. 1:**
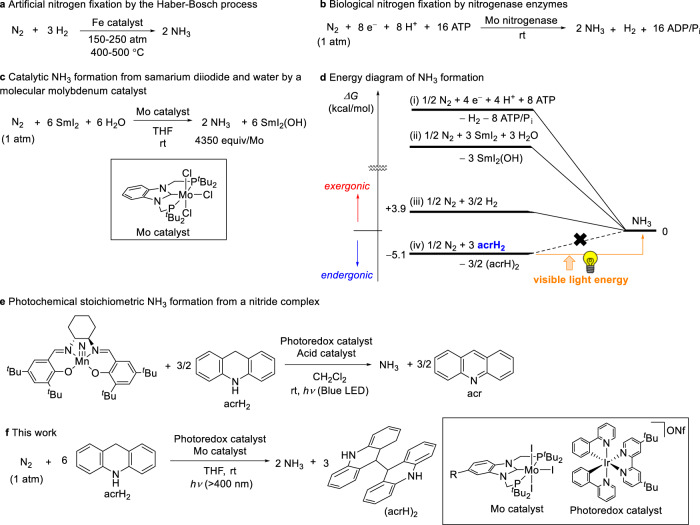
Nitrogen fixation by chemical and visible light energy. **a** The industrial ammonia production from dinitrogen and dihydrogen by the Haber–Bosch process. **b** The biological reduction of dinitrogen into ammonia with a reductant, a proton source, and ATP by Mo nitrogenase. **c** Catalytic ammonia formation in the presence of the molybdenum complex using SmI_2_ and water. **d** Energy profiles of the formation of ammonia by (i) nitrogenase, (ii) our previous molybdenum catalytic system, (iii) N_2_ and H_2_ under the standard state and (iv) this work. **e** Stoichiometric transformation of a manganese–nitride complex into ammonia using acrH_2_ in the presence of a photoredox catalyst. **f** The current reaction system for catalytic ammonia formation using visible light energy.

The conversion of atmospheric dinitrogen into highly useful compounds like ammonia, the so-called nitrogen fixation process, is one of the most important reactions for human beings. Currently, ammonia is produced industrially from dinitrogen and dihydrogen using heterogeneous catalysts under harsh reaction conditions in the Haber–Bosch process (Fig. 1a)^1^. Although the free energy change associated with ammonia formation is negative in the standard state, high reaction temperatures are required to cleave the N ≡ N bond, resulting in the need for high operating pressures. Given that ammonia is one of the most industrially important products, the amount of this compound produced yearly worldwide via the Haber–Bosch process has reached 180 million tonnes^2^. Notably, in this process, dihydrogen is derived from fossil fuels and carbon dioxide is emitted as a side-product^3,4^. Therefore, the development of a synthetic approach to the production of ammonia, which does not involve the use of fossil fuels, is particularly desirable from the standpoint of sustainability.

In contrast to the Haber–Bosch process, the nitrogen-fixing enzymes called nitrogenases produce in nature ammonia from dinitrogen and water under ambient reaction conditions (Fig. 1b)^5^. In the case of Mo nitrogenase, the formation of two molecules of ammonia accompanies the hydrolysis of sixteen molecules of ATP, a process that supplies the energy that drives the reaction, consuming a lot of energy^6,7^. Recent studies have provided indication that the active site of the nitrogenase contains sulphur-bridged clusters that include transition metals^8–10^. The synthesis of transition metal clusters that mimic the structure of the active site of nitrogenase and promote the relevant stoichiometric reactions have been intensively investigated to reproduce the function of the nitrogenases^11–14^. On the other hand, the synthesis and reactivity of transition metal–nitrogen complexes, wherein dinitrogen is coordinated to the metal centre, are deemed model reactions for nitrogenase-catalysed processes, so they have been the subject of intensive research^15–20^.

In 2003, Yandulov and Schrock reported the first successful example of transition metal–catalysed ammonia formation from dinitrogen achieved using reducing reagents and proton sources under ambient reaction conditions^21^. Since then, catalytic reactions for ammonia formation under mild reaction conditions have been realised using various transition metal complexes^13–20^. In 2019, our research group developed an ammonia production system operating under ambient conditions wherein samarium iodide and water acted as the reductant and the proton source, respectively (Fig. 1c)^22^. In this reaction system, molybdenum complexes bearing an *N*-heterocyclic carbene-based PCP-type pincer ligand worked as the most effective catalyst to afford the production of up to 4350 equiv. of ammonia, based on the number of molybdenum atoms of the catalyst whose use was associated with the highest turnover frequency.

Notably, the reaction of ammonia formation catalysed by transition metal complexes described in the previous section is not ideal, because the driving force of ammonia production is provided by the energy derived from the chemical reagents (Fig. 1d)^19^. By contrast, in an ideal reaction, ammonia production is not driven by chemical energy but by some form of renewable energy. Therefore, the development of a catalytic ammonia production process that relies on the energy provided by visible light is an important research goal from the viewpoint of sustainable chemistry. In 2019, Chirik, Knowles and co-workers reported the photoredox-catalysed reaction of a manganese–nitride complex with 9,10-dihydroacridine (acrH_2_) at room temperature to afford a stoichiometric amount of ammonia, based on the number of complex-based manganese atoms (Fig. 1e)^23^. In this reaction system, acrH_2_ acted as both reductant and proton source under visible light irradiation to release two electrons and two protons. Chirk and co-workers also found photochemical hydrogenation of the manganese-nitride complex to give a stoichiometric amount of ammonia^24^. Quite recently, Chirik and co-workers reported the stoichiometric formation of ammonia from the iridium-catalysed hydrogenation of a molybdenum-nitride complex, which was generated from dinitrogen, under photoirradiation and ambient reaction conditions^25^.

Against this research backdrop, we envisage the photoredox- and molybdenum-catalysed reduction of dinitrogen into ammonia whereby acrH_2_ acts as both reductant and proton source under visible light irradiation (Fig. 1f). Since the formation of ammonia as a result of the reaction of dinitrogen with acrH_2_ is calculated to be endergonic (see below), visible light provides the driving force for the described process (Fig. 1d). We believe that the results of the present study point to the manufacture of an unprecedented and revolutionary reaction system for ammonia synthesis that is operational under ambient reaction conditions.

## Results

### Catalytic ammonia formation

**Fig. 2:**
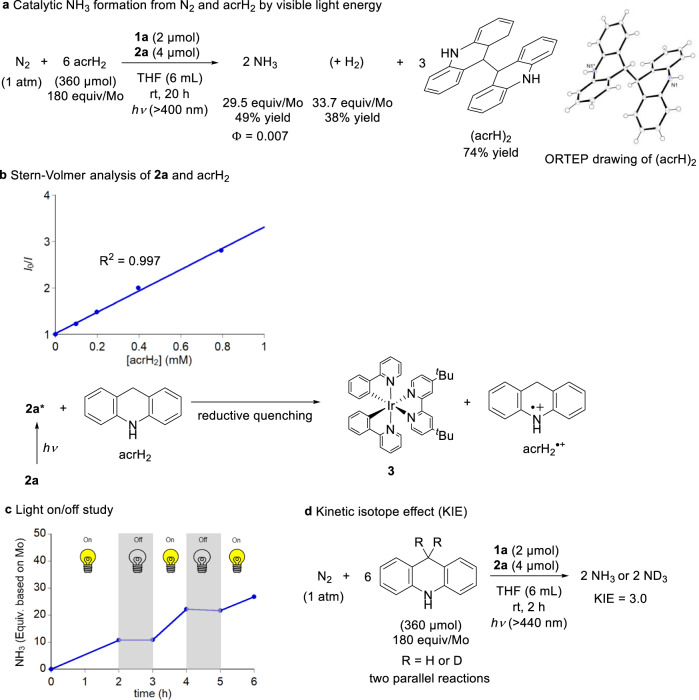
Catalytic nitrogen fixation using visible light. **a** Ammonia formation from dinitrogen and acrH_2_ in the presence of molybdenum complex **1a** and photoredox catalyst **2a**. **b** Emission quenching experiment of photoredox catalyst **2a** with acrH_2_ to form the reduced iridium catalyst (**3**) and acrH_2_^•+^. **c** Light on/off experiment. **d** Kinetic isotope effect (KIE) on the visible light driven ammonia formation.

The reaction of dinitrogen at atmospheric pressure with 180 equiv. of acrH_2_ in the presence of catalytic amounts of a molybdenum triiodide complex bearing the PCP-type pincer ligand [MoI_3_(PCP)]^26^(**1a**: PCP = 1,3-bis((di-*tert*-butylphosphino)methyl)benzimidazol-2-ylidene) and of [Ir(ppy)_2_(dtbbpy)]ONf (**2a**: ppy = 2-(2-pyridyl)phenyl; dtbbpy = 4,4′-di-*tert*-butyl-2,2′-bipyridine; ONf = OSO_2_C_4_F_9_, nonafluorobutanesulfonate) acting as a photoredox catalyst in tetrahydrofuran (THF) at room temperature for 20 h under visible light irradiation afforded 29.5 equiv. of ammonia, based on the number of catalyst-based molybdenum atoms (49% yield), alongside 33.7 equiv. of dihydrogen (38% yield) (Fig. 2a and Table 1, Entry 1). Initially, we assumed that, under visible light irradiation, acrH_2_ acted as a two-electron and two-proton source, so that it underwent conversion into acridine (acr)^23^. Interestingly, however, contrary to our expectations, under the implemented reaction conditions, acrH_2_ acted as a one-electron and one-proton source. In fact, after the catalytic reaction, we observed the formation of 9,9′,10,10′-tetrahydro-9,9′-biacridine ((acrH)_2_) in 74% yield (Fig. 2a), while we did not observe any acr being formed. We assume that (acrH)_2_ was produced via the homo-coupling reaction of a radical intermediate generated as a result of the oxidation and deprotonation of acrH_2_. The thus produced (acrH)_2_ was characterised by proton nuclear magnetic resonance (^1^H NMR) spectroscopy and X-ray analysis. Indeed, an ORTEP drawing of (acrH)_2_ is shown in Fig. 2a.

**Table 1:** Visible light driven catalytic nitrogen fixation using molybdenum catalysts and photoredox catalysts

Entry	Mo catalyst	Photoredox catalyst	NH_3_ production (equiv. based on Mo)	NH_3_ yield (%)^a^	H_2_ production (equiv. based on Mo)	H_2_ yield (%)^a^
1	**1a**	**2a**	29.5 ± 1.3	49.1 ± 2.3	33.7 ± 0.3	37.5 ± 0.3
2	**1b**	**2a**	0.5	0.8	5.7	6.3
3	**1c**	**2a**	3.0	5.0	61.7	68.6
4	**1d**	**2a**	39.8 ± 3.1	66.2 ± 5.2	21.3 ± 7.2	23.6 ± 8.0
5^b^	**1d**	**2a**	41.3 ± 6.2	34.4 ± 5.2	12.0 ± 1.7	6.7 ± 0.9
6	**1a**	**2b**	0.4	0.6	2.5	2.8
7	**1a**	**2c**	17.6	29.3	18.1	20.1
8	**1a**	**2d**	10.0	16.6	13.3	14.7
9	**1a**	**2e**	5.5	9.2	11.2	12.4
10^c^	**1a**	**2a**	0.2	0.4	58.6	65.1
11^d^	**1a**	**2a**	0.5	—	0	—
12^e^	**1a**	**2a**	0.1	0.2	0	0
13	none	**2a**	—	0.3	—	0
14	**1a**	none	0.3	0.6	0.2	0.2
15	**1e**	**2a**	15.2	25.3	8.8	9.8

^a^Yield based on acrH_2_. ^b^acrH_2_ (720 μmol, 360 equiv./Mo) was used. ^c^Under Ar atmosphere (1 atm). ^d^Without acrH_2_. ^e^Dark condition.

**Fig. 3:**
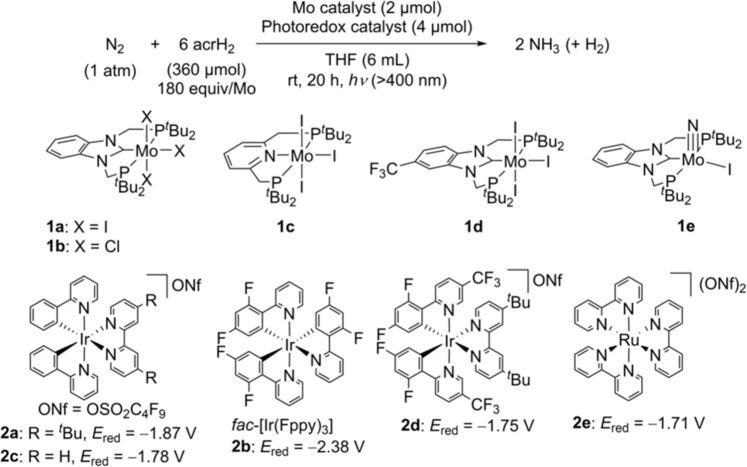
Visible Light Driven Catalytic Nitrogen Fixation Using Molybdenum Catalysts and Photoredox Catalysts. Reduction potentials of photoredox catalysts in MeCN are taken from refs. 30–32. *E*_red_ is a potential of Ir(II/III) or Ru(I/II).

The nature of the molybdenum and photoredox catalysts has a decisive influence on the catalytic reaction. The use as catalysts of other molybdenum complexes, such as a molybdenum trichloride complex bearing the PCP-type pincer ligand [MoCl_3_(PCP)]^27^ (**1b**) anda molybdenum triiodide complex bearing a pyridine-based PNP-type pincer ligand [MoI_3_(PNP)]^28^ (**1c**: PNP = 2,6-bis(di-*tert*-butylphosphinomethyl)pyridine), afforded the production of only a small amount of ammonia (Fig. 3 and Table 1, Entries 2 and 3). By contrast, a molybdenum triiodide complex bearing a trifluoromethyl-substituted PCP-type pincer ligand [MoI_3_(CF_3_-PCP)]^29^ (**1d**; see structure in Fig. 3) worked as a more effective catalyst than **1a**; indeed, in the presence of this complex, up to 39.8 equiv. of ammonia were produced based on the number of catalyst-based molybdenum atoms (66% yield), together with 21.3 equiv. of dihydrogen (24% yield) (Table 1, Entry 4). Conducting the reaction in the presence of 360 equiv. of acrH_2_ (instead of 180 equiv.), under the same reaction conditions, resulted in a slightly higher amount of ammonia observed to be produced based on the amount of the catalyst (Table 1, Entry 5). On the other hand, the use of photoredox catalysts with a suitable reducing ability is necessary to promote the catalytic reaction effectively. When *fac*-[Ir(Fppy)_3_] (**2b**; see structure in Fig. 3), which is characterised by a higher reducing ability than **2a**, was used as a photoredox catalyst in place of **2a**, only 0.4 equiv. of ammonia were produced, based on the number of catalyst-based molybdenum atoms (Table 1, Entry 6). When photoredox catalysts **2c**–**2e** (see structures in Fig. 3), which exhibit a lower reducing ability than **2a**, were utilised in the ammonia production process, the amount of ammonia produced was observed to decrease as the reducing ability of the photoredox catalyst decreased (Table 1, Entries 7–9).

The reaction solvent was also determined to play an important role in promoting the catalytic reaction. When toluene, dimethyl sulfoxide (DMSO), 1,4-dioxane and dichloromethane were employed as solvents, only a small amount of ammonia was obtained, based on the number of catalyst-based molybdenum atoms. However, a moderate amount of ammonia was observed to be produced when dimethoxyethane (DME) was used as solvent (see the Supplementary Information). The results of several control experiments indicate that the combination of dinitrogen, acrH_2_, visible light, a molybdenum complex and an iridium complex is essential to promote the catalytic formation of ammonia (Table 1, Entries 10–14). Separately, we confirmed the direct conversion of molecular dinitrogen to ammonia when **1a** was used as a catalyst in a reaction conducted under atmospheric pressure of ^15^N_2_ gas, in place of an atmospheric pressure of regular ^14^N_2_ gas (see the Supplementary Information).

### Mechanistic Investigation

The reduction potential of **2a** was determined to be *E*^1/2^_red_ = −1.88 V versus FeCp_2_^+/0^ (Cp = *η*^5^-C_5_H_5_) by cyclic voltammetry (Supplementary Fig. 1); moreover, the value for this compound’s excitation energy (*E*_0_) has been reported to be 2.17 eV;^30–32^ thus, the reduction potential of the excited state of **2a** (2a*) was estimated to be *E*_red_* = +0.29 V vs. FeCp_2_^+/0^. The oxidation peak potential of acrH_2_ was *E*_pa_ = +0.41 V vs. FeCp_2_^+/0^ (Supplementary Fig. 2). Even though the electron transfer from acrH_2_ to 2a* can be estimated to be a slightly endergonic process, based on the mentioned values, the results of a herein-conducted Stern–Volmer analysis for emission quenching of **2a** by acrH_2_ indicated the linear plots with 2.3 mM^−1^ of the Stern–Volmer constant (*K*_SV_) (Fig. 2b). This observation points to a situation whereby the reduction of **2a** by acrH_2_ can proceed via a photo-induced single-electron transfer process.

**Fig. 4:**
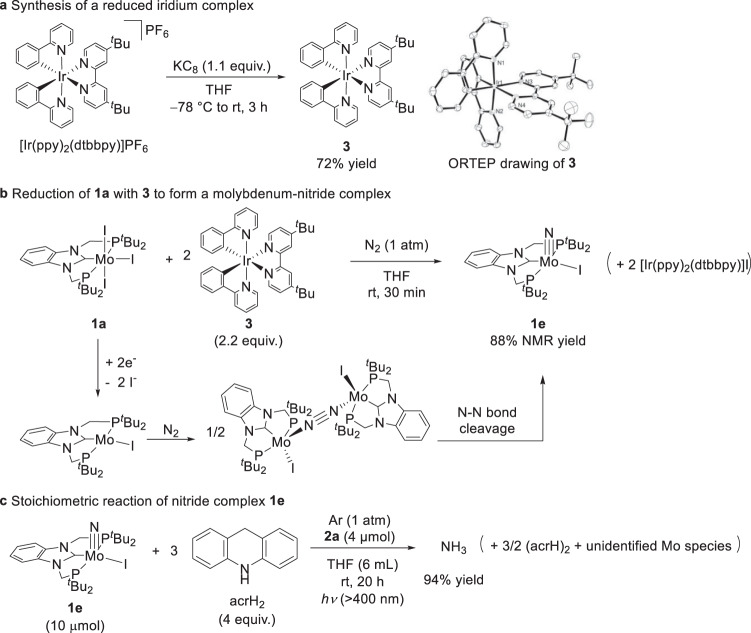
Stoichiometric reactions of iridium and molybdenum complexes. **a** Reduction of iridium complex [Ir(ppy)_2_(dtbbpy)]PF_6_ into [Ir(ppy)_2_(dtbbpy)] (**3**). **b** Formation of molybdenum–nitride complex (**1e**) from the reaction of **1a** with **3** under N_2_. **c** Transformation of the nitride ligand in **1e** to ammonia using acrH_2_ in the presence of a photoredox catalyst **2a** under visible light irradiation.

In order to confirm whether the subsequent stage of the ammonia production process is triggered by the formation of the one-electron-reduced derivative of **2a**, we directly synthesised the one-electron-reduced derivative of **2a**; in other words, we prepared the neutral complex [Ir(ppy)_2_(dtbbpy)] (**3**) using a chemical reductant. Specifically, [Ir(ppy)_2_(dtbbpy)]PF_6_ was reduced with 1.1 equiv. of KC_8_ in THF at −78 °C to room temperature for 3 h (Fig. 4a). The detailed molecular structure of **3** was confirmed by X-ray analysis (Fig. 4a). Cyclic voltammetry experiments conducted on **3** indicated that the system’s oxidation wave in THF appeared at −1.87 V versus FeCp_2_^+/0^ and that the relevant process was reversible (Supplementary Fig. 3). This evidence indicates that **3** exhibits a reducing ability that is comparable to that of decamethylcobaltocene (CoCp*_2_; Cp* = *η*^5^-C_5_Me_5_), which was employed as a reductant in our previously published study focusing on ammonia formation^28^.

Next, we conducted a reaction between **1a** and 2.2 equiv. of **3** in THF at room temperature for 30 min under an atmospheric pressure of dinitrogen; as a result, the corresponding molybdenum–nitridecomplex [Mo(≡N)I(PCP)]^28^ (**1e**) was obtained in 88% yield, as determined by NMR spectroscopy (Fig. 4b). Evidence thus indicates that a dinitrogen-bridged dimolybdenum complex is formed followingthe two-electron reduction of **1a**; moreover, cleavage of the N ≡ N bond in the dinitrogen-bridged complex affords the corresponding molybdenum–nitride complex **1e**. The further reaction of **1e** with 4 equiv. of acrH_2_ in the presence of **2a** acting as a photoredox catalyst under 1 atm of argon gas and visible light irradiation afforded ammonia in 94% yield, based on the number of the complex-based molybdenum atoms (Fig. 4c). Additionally, the nitride complex **1e** acted as catalyst under the standard reaction conditions (Table 1, Entry 15). This result indicates that the present reaction pathway proceeds via the splitting route (see below) previously proposed by our research group^28,33^.

A light on/off experiment was also conducted for the reaction with acrH_2_ under the typical catalytic reaction conditions; its results indicated that ammonia formation ceased completely in the dark, suggesting that a chain propagation is not the main reaction pathway and that continuous irradiation with visible light is necessary for the reaction to proceed (Fig. 2c). When an Hg lamp with a 410 nm band pass filter (Kenko B410) was utilised as the light source, the apparent quantum yield (Φ) of ammonia formation with acrH_2_ under typical reaction conditions was determined by chemical actinometry to have a value of 0.007 (Fig. 2a). Both the results of the light on/off experiment and the quantum yield measurement indicate that ammonia formation did not proceed via a radical chain process. The catalytic reaction was also conducted under the typical conditions using 9,9-dideuterio-9,10-dihydroacridine as reductant, so as to estimate the kinetic isotope effect (KIE). The ratio of the reaction rates of ammonia formation measured in two parallel reactions involving the hydrogenated and deuterated reductants was 3.0 (Fig. 2d), which suggests that this catalytic reaction comprises a proton-coupled electron transfer (PCET)^34^ or a proton transfer reaction involving acrH_2_.

### Proposed reaction pathway for catalytic ammonia formation

**Fig. 5:**
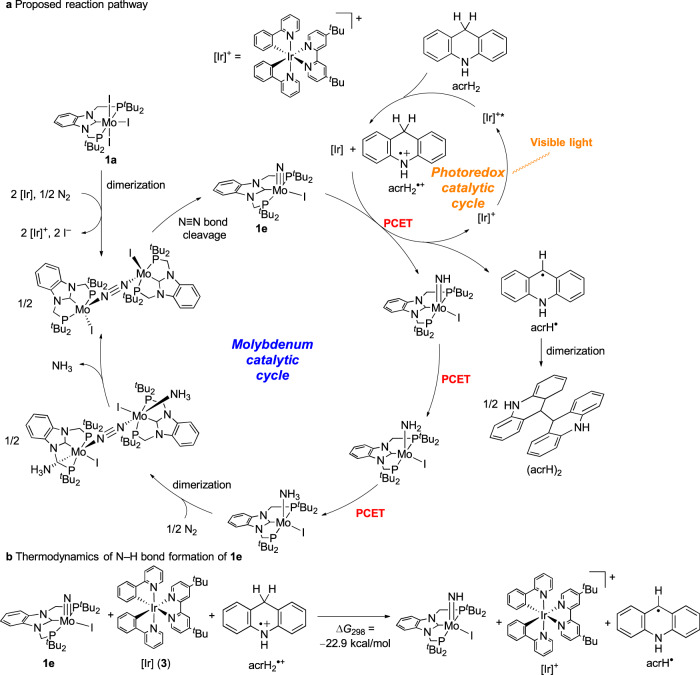
Possible reaction pathway of molybdenum- and photoredox-catalysed ammonia formation. **a** Proposed reaction pathway of reduction of dinitrogen into ammonia using visible light energy by molybdenum- and photoredox cycles. PCET = proton-coupled electron transfer. **b** Calculated free energy change of the formation of the imide complex from the nitride complex **1e** with [Ir] **3** and acrH_2_^•+^.

A plausible reaction pathway for the cooperative photoredox- and molybdenum-catalysed reduction of dinitrogen with acrH_2_ is shown in Fig. 5a. This pathway comprises two catalytic cycles: the photoredox catalytic cycle and the molybdenum catalytic cycle. In the photoredox catalytic cycle, the iridium catalyst [Ir]^+^ is excited under visible light irradiation to produce a photoexcited iridium catalyst [Ir]^+^*. Subsequently, a single-electron transfer process takes place between [Ir]^+^* and acrH_2_, which produces the reduced iridium catalyst [Ir] and the radical cationic 9,10-dihydroacridine (acrH_2_^•+^). An electron transfer from [Ir] and a proton transfer from acrH_2_^•+^ to the molybdenum–nitride complex [Mo(≡N)I(PCP)] (**1e**), which is formed as a result of the reduction of [MoI_3_(PCP)] (**1a**) by [Ir] under dinitrogen atmosphere, simultaneously occur as a PCET process to afford the molybdenum–imide complex [Mo(=NH)I(PCP)] together with [Ir]^+^ and the corresponding radical species (acrH^•^), which dimerises to form (acrH)_2_ as a precipitate. Low solubility of (acrH)_2_ in THF might provide driving force for this PCET step. However, we cannot exclude the possibility of a stepwise process of protonation and reduction because protonation of **1e** with acrH_2_^•+^ would proceed in an exergonic way (Supplementary Fig. 22). Similar PCET or protonation-reduction processes take place to afford the molybdenum–ammonia complex [Mo(–NH_3_)I(PCP)] after the formation of the molybdenum–amide complex [Mo(–NH_2_)I(PCP)]. Subsequently, following the formation of the dinitrogen-bridged dimolybdenum–ammonia complex, the dissociation of the ammonia ligand from the complex takes place. Finally, the dinitrogen-bridged dimolybdenum complex is converted into the starting molybdenum–nitride complex [Mo(≡N)I(PCP)] via direct cleavage of the bridging dinitrogen ligand of the dinitrogen-bridged dimolybdenum complex as the splitting route.

In order to obtain additional information on the proposed reaction pathway, we carried out density functional theory calculations. The computational evidence thus collected indicates that the reaction of [Mo(≡N)I(PCP)] **1e** with [Ir] **3** and acrH_2_^•+^ to produce [Mo(=NH)I(PCP)], the [Ir]^+^ complex and acrH^•^ proceeds smoothly, and it is characterised by a free energy change at 298 K (Δ*G*_298_) of −22.9 kcal/mol (Fig. 5b). The generated Mo-imide complex should be transformed into the corresponding amide and ammonia complexes more smoothly, because the imide complex has the smallest bond dissociation free energy (BDFE) of the N–H bond of [Mo(NH_*x*_)I(PCP)] (*x* = 1–3), where 34 kcal/mol (*x* = 1), 53 kcal/mol (*x* = 2), and 41 kcal/mol (*x* = 3)^29^. These calculated results support the view that the reduced photoredox catalyst [Ir] **3** and the radical cation acrH_2_^•+^ act as electron and proton sources, respectively, in the PCET process to form the N–H bonds.

With respect to the role of acrH_2_ in our reaction system, as can be evinced from the reaction scheme shown in Fig. 2a, acrH_2_ was transformed exclusively into (acrH)_2_ via the homo-coupling reaction of radical intermediate acrH^•^ generated via oxidation and deprotonation of acrH_2_. Therefore, acrH_2_ acts as sacrificial one-electron and one-proton source in the present reaction. The fact that no acr forms in our reaction system indicates that acrH^•^ does not act as a hydrogen atom (or one-electron/one-proton) source under the reaction conditions applied in this study. Indeed, our experimental results are in sharp contrast with those reported by Chirik, Knowles and co-workers^23^, who found that acr was selectively formed as a result of the reaction of the manganese–nitride complex with acrH_2_ in the presence of a photoredox catalyst (Fig. 1e). We assume that the unique reactivity ofacrH_2_ in our reaction system is due to the values of the BDFEs of the N–H bonds of the molybdenum–imide, molybdenum–amide and molybdenum–ammonia complexes (34, 53, and 41 kcal/mol, respectively)^29^ being smaller than those of the corresponding manganese complexes (60, 84, and 85 kcal/mol, respectively)^23^, which are large enough to drive the transfer of the hydrogen atom from acridine species like acrH_2_, acrH_2_^•+^ and acrH^•^ to the manganese nitrogenous complexes^23^.

Finally, we evaluated the present reaction system from the viewpoint of thermodynamics. The reaction between dinitrogen and acrH_2_ to form ammonia and (acrH)_2_ is calculated to be an endergonic process, with a value for the change in Gibbs free energy of 5.1 kcal/mol (Eq. 1 and Fig. 1d). This evidence indicates that the described reaction does not proceed spontaneously, and it is driven by the energy of the irradiated visible light. In other words, the use of visible light renders possible the described thermally-prohibited transformation.

1 $$1/2\,{{{{{{\rm{N}}}}}}}_{2}+3\,{{{{{{\rm{acrH}}}}}}}_{2}\to {{{{{{\rm{NH}}}}}}}_{3}+3/2 \, {({{{{{\rm{acrH}}}}}})}_{2}$$

## Discussion

In summary, we have identified a process whereby ammonia is formed from the reaction of dinitrogen with acrH_2_ acting as a formal hydrogen source in the presence of both iridium and molybdenum complexes acting as catalysts under ambient reaction conditions and visible light irradiation. Detailed investigations indicated that, under the applied reaction conditions, acrH_2_ acted as a one-electron and one-proton source. The results described in the present manuscript represents the first successful example of the visible light-enabled thermally-prohibited catalytic ammonia formation from dinitrogen with transition metal complexes under ambient reaction conditions. Generation of (acrH)_2_ as a stoichiometric waste needs to be solved toward development of sustainable ammonia synthesis. However, we believe that the results of the present study represent a research breakthrough with respect to the process whereby visible light energy is utilised to catalytically convert molecular nitrogen to ammonia, which can in turn be employed as an energy source.

## Methods

### General information

Detailed experimental procedures, characterization of compounds and the computational details can be found in the Supplementary Methods, Supplementary Figs. 1–22, and Supplementary Tables 1–9. Cartesian coordinates are available in Supplementary Data 1.

### Catalytic ammonia formation under visible light irradiation

A typical experimental procedure for the catalytic reactions is described below. In a 50 mL Schlenk flask were placed molybdenum catalyst (0.0020 mmol), acrH_2_ (0.36 mmol), and photocatalyst (0.0040 mmol). The Schlenk flask was evacuated and then filled with N_2_. THF (6 mL) was added to the flask, and the mixture was irradiated (>400 nm) with stirring at room temperature for 20 h. After the reaction, the amount of generated dihydrogen was quantified by GC. Then, an aqueous potassium hydroxide solution (30 wt%, 5 mL) was added to the reaction mixture. The mixture was evaporated under reduced pressure, and the distillate was trapped in a dilute H_2_SO_4_ solution (0.5 M, 10 mL). The amount of ammonia present in the H_2_SO_4_ solution was determined by the indophenol method. No hydrazine was detected by the *p*-(dimethylamino)benzaldehyde method.

## Supplementary information

Supplementary Information

Supplementary Data 1

## Data Availability

The X-ray crystallographic coordinates for structures reported in this article have been deposited at the Cambridge Crystallographic Data Centre (CCDC), under deposition number CCDC 2189300 ((acrH)_2_) and 2189301 (**3**·2 C_4_H_8_O). These data can be obtained free of charge from The Cambridge Crystallographic Data Centre via www.ccdc.cam.ac.uk/data_request/cif. Cartesian coordinates are available in Supplementary Data 1. All other data are available from the authors upon reasonable request.
