# Supplementary material for: Catalytic nitrogen fixation using visible light energy
Source: Nat Commun. 2022 Dec 1;13:7263. doi: 10.1038/s41467-022-34984-1 (PMC9715552; doi:10.1038/s41467-022-34984-1)
Supplement: Supplementary file 1 — Supplementary Information [file 41467_2022_34984_MOESM1_ESM.pdf]

Supplementary Information

Catalytic nitrogen fixation using visible light energy

Yuya Ashida[1], Yuto Onozuka[1], Kazuya Arashiba[1], Asuka Konomi[2], Hiromasa Tanaka[3], Shogo Kuriyama[1], Yasuomi Yamazaki[1], Kazunari Yoshizawa[2]*, Yoshiaki Nishibayashi[1]*

[1]Department of Applied Chemistry, School of Engineering, The University of Tokyo, Bunkyo-ku, Tokyo 113-8656, Japan
[2]Institute for Materials Chemistry and Engineering, Kyushu University, Nishi-ku, Fukuoka 819-0395, Japan
[3]School of Liberal Arts and Sciences, Daido University, Minami-ku, Nagoya 457-8530, Japan

*Correspondence and requests for materials should be addressed to K.Y. (Email: kazunari@ms.ifoc.kyushu-u.ac.jp) and Y.N. (Email: ynishiba@g.ecc.u-tokyo.ac.jp).

Table of Contents

1. Supplementary methods S2
- **1.1 General methods** S2
- **1.2 Experimental procedures** S2
- **1.3 Cyclic voltammetric studies** S9
- **1.4 NMR spectra** S12
- **1.5 UV-vis spectra** S15
- **1.6 Light ON/OFF experiments** S17
- **1.7 Stern-Volmer analysis** S18
- **1.8 Determination of quantum yield** S19
- **1.9 X-ray crystallographic studies** S20
- **1.10 Computational Details** S24

2. Supplementary references S29

1. Supplementary methods

1.1 General methods.

^{1}H NMR (400 MHz), $^{31}P\{^{1}H\}$ NMR (162 MHz) and $^{15}N\{^{1}H\}$ NMR (41 MHz) spectra were recorded on a JEOL ECS-400 spectrometer in suitable solvent, and spectra were referenced to residual solvent (^{1}H) or external standard ($^{31}P\{^{1}H\}$: 85% H_3PO_4, $^{15}N\{^{1}H\}$: CH_3NO_2). Photoirradiation was conducted using mercury lamp USH 250SC2 (250 W) with a sharp cut filter (Schott, GG400, $\lambda > 400$ nm). Absorption spectra were recorded on a Shimadzu UV-1850. Photoluminescence spectra were measured on a Shimadzu RF-5300PC spectrophotometer. Evolved dihydrogen was quantified by gas chromatography (GC) using a Shimadzu GC 8A equipped with a thermal conductivity detector and a ShinCarbon ST column (6 m × 3 mm). Elemental analyses were performed at Microanalytical Center of The University of Tokyo.

All manipulations were carried out under an atmosphere of nitrogen or argon by using standard Schlenk techniques or glovebox techniques unless otherwise stated. Solvents were dried by general methods and degassed before use. [MoX_3(PCP)] (**1a**: X = I[1], **1b**: X = Cl[2], PCP = 1,3-bis((di-*tert*-butylphosphino)methyl)benzimidazol-2-ylidene)), [MoI_3(PNP)] (**1c**, PNP = 2,6-bis(di-*tert*-butylphosphinomethyl)pyridine)),[3] (CF_3-PCPH)PF_6 (CF_3-PCPH = 1,3-bis((di-*tert*-butylphosphino)methyl)-6-(trifluoromethyl)-1*H*-benzo[*d*]imidazol-3-ium),[4] **2a**,[5] **2c**,[5] **2d**,[5] **2e**,[5] $MoI_3(thf)_3$ (thf = tetrahydrofuran),[6] and $CoCp^*_2$ (Cp* = η^5-C_5Me_5)[7] were prepared according to the literature methods. Other reagents were purchased commercially and used as received. Other reagents were purchased commercially and used as received.

1.2 Experimental procedures.

Synthesis of [$MoI_3(CF_3$-PCP)] (1d). To a cooled solution of (CF_3-PCPH)PF_6 (388 mg, 0.60 mmol) in toluene (15 mL) at -78 °C was added $KN(SiMe_3)_2$ (133 mg, 0.67 mmol), and the mixture was stirred at room temperature for 1 h. The mixture was filtered through Celite, and the filter cake was washed with toluene (1 mL x 3). The solvent was removed under reduced pressure to give a yellow-brown oily solid. To the residue were added [$MoI_3(thf)_3$] (347 mg, 0.50 mmol) and THF (25 mL), and the mixture was stirred at 50 °C for 16 h. The resultant dark brown solution was filtered, and slow addition of hexane (40 mL) to the filtrate afforded **1d**·THF (383 mg, 0.36 mmol, 73%) as a brown crystalline solid. Magnetic susceptibility of **1d** was measured using the Evans method:[8] μ_{eff} = 3.5 μ_B in THF-d_8 at 295 K. Anal. Calcd. for $C_{26}H_{43}F_3I_3MoN_2P_2$ + C_4H_8O: C, 34.27; H, 4.89; N, 2.66. Found: C, 34.59; H, 4.89; N, 2.74.

Synthesis of [Mo(N)I(PCP)] (1e). To a solution of [MoI_3(PCP)] (274 mg, 0.30 mmol) in THF (15 mL) was added a solution of $CoCp^*_2$ (217 mg, 0.66 mmol) in THF (15 mL) at -78 °C, and the mixture was stirred at room temperature for 18 h. The resultant yellow-brown suspension was filtered through Celite, and the filter cake was washed with THF (2 mL × 3). Volatiles were removed in vacuo, and the residue was washed with hexane (5 mL × 3) and dried in vacuo. The resultant yellow-brown solid was extracted with THF (30 mL), and slow addition of hexane (30 mL) to the extract afforded **1e** as yellow-brown crystals, which were collected by filtration, washed with hexane (2 mL × 3), and dried in vacuo (120 mg, 0.18 mmol, 60%). ^{1}H NMR (THF-d_8): δ 7.71 (s, 2H), 7.40 (s, 2H), 4.80 (d, J = 12.8 Hz, 2H), 4.66 (d, J = 12.8 Hz, 2H), 1.61 (s, 18H), 1.17 (s, 18H). ^{31}P{^{1}H} NMR (THF-d_8): δ 113.3 (s). Anal Calcd for $C_{25}H_{44}IMoN_3P_2$+C_4H_8O: C, 46.84; H, 7.05; N, 5.65. Found: C, 47.12; H, 7.01; N, 5.76. Spectroscopic data were identified with previously reported literature.[4]

General procedures for catalytic ammonia formation under visible-light irradiation. A typical experimental procedure for the catalytic reactions is described below. In a 50 mL Schlenk flask were placed molybdenum catalyst (0.0020 mmol), 9,10-dihydroacridine (0.36 mmol), and photocatalyst (0.0040 mmol). The Schlenk flask was evacuated and then filled with N_2. THF (6 mL) was added to the flask, and the mixture was irradiated (> 400 nm) with stirring at room temperature for 20 h. After the reaction, the amount of generated dihydrogen was quantified by GC. Then, an aqueous potassium hydroxide solution (30 wt%, 5 mL) was added to the reaction mixture. The mixture was evaporated under reduced pressure, and the distillate was trapped in a dilute H_2SO_4 solution (0.5 M, 10 mL). The amount of ammonia present in the H_2SO_4 solution was determined by the indophenol method.[9] No hydrazine was detected by the p-(dimethylamino)benzaldehyde method.[10] The control experiments are shown in **Supplementary Table 1**. The results using various solvents, photocatalysts, and molybdenum catalysts are shown in **Supplementary Tables 2-4**.

Supplementary Table 1. Catalytic ammonia formation using visible light: control experiments.

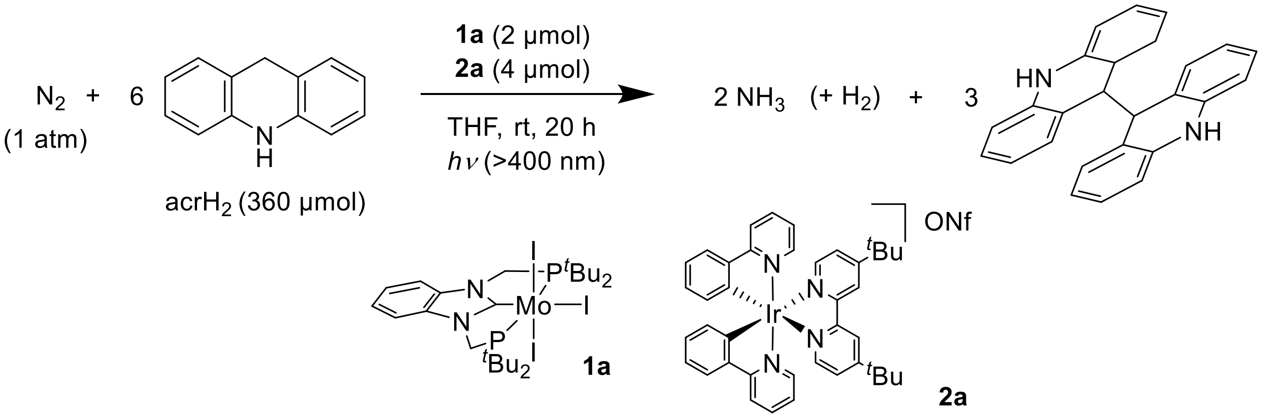

Entry	Conditions	NH_3 (equiv/Mo)	NH_3 (%)[a]	H_2 (equiv/Mo)	H_2 (%)[a]
1	-	29.5 ± 1.3	49.1 ± 1.3	33.7 ± 0.3	37.5 ± 0.4
2	without $acrH_2$	0.5	-	0	-
3	without Mo cat.	-	0.3	-	0
4	without photocat.	0.3	0.6	0.2	0.2
5	without light	0.1	0.2	0	0
6	under Ar	0.2	0.4	58.6	65.1

[a]Yield based on $acrH_2$.

Supplementary Table 2. Catalytic ammonia formation using visible light: solvents.

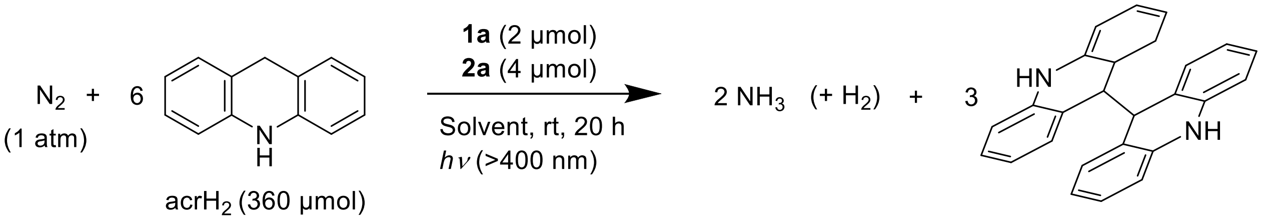

Entry	Solvent	NH_3 (equiv/Mo)	NH_3 (%)[a]	H_2 (equiv/Mo)	H_2 (%)[a]
1	THF	29.5 ± 1.3	49.1 ± 1.3	33.7 ± 0.3	37.5 ± 0.4
2	toluene	2.2	3.6	0.6	0.7
3	DMSO	0	0	0	0
4	1,4-dioxane	0.5	0.8	16.9	18.7
5	DME	13.6	22.6	38.1	42.3
6	CH_2Cl_2	0	0	0	0

[a]Yield based on $acrH_2$.

Supplementary Table 3. Catalytic ammonia formation using visible light: photoredox catalysts.

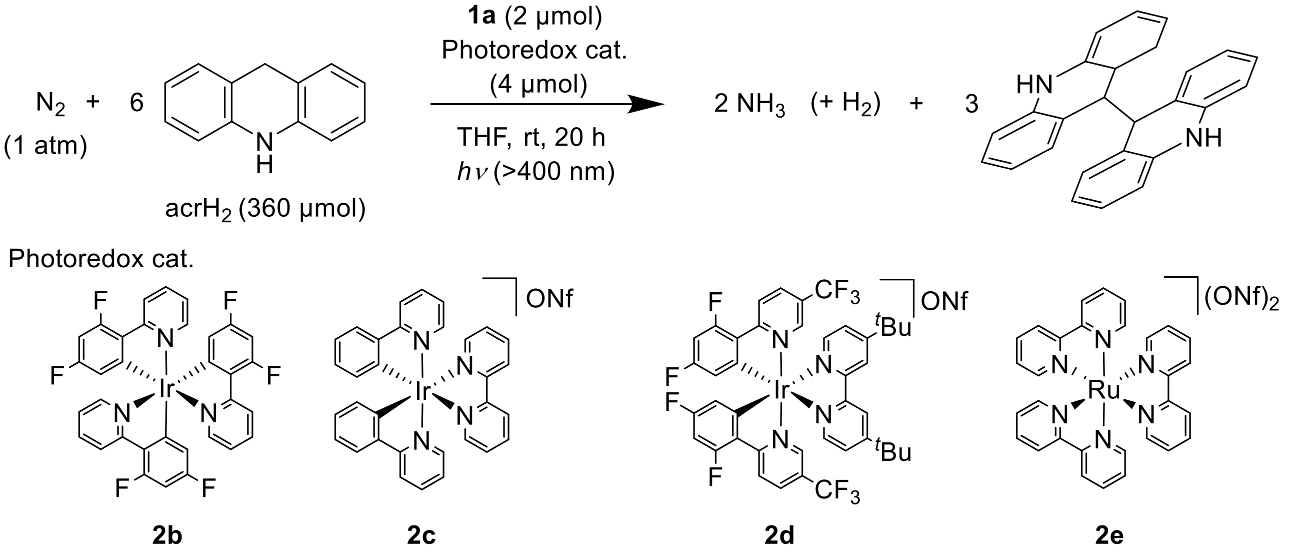

Entry	Photoredox cat.	E_{red} (V)[a]	NH_3 (equiv/Mo)	NH_3 (%)[b]	H_2 (equiv/Mo)	H_2 (%)[b]
1	[Ir(ppy)$_2$(dtbbpy)]ONf (**2a**)	-1.89	29.5 ± 1.3	49.1 ± 1.3	33.7 ± 0.3	37.5 ± 0.4
2	*fac*-Ir(Fppy)$_3$ (**2b**)	-2.38	0.4	0.6	2.5	2.8
3	[Ir(ppy)$_2$(bpy)]ONf (**2c**)	-1.78	17.6	29.3	18.1	20.1
4	[Ir(dF(CF$_3$)ppy)$_2$(dtbbpy)]ONf (**2d**)	-1.75	10.0	16.6	13.3	14.7
5	[Ru(bpy)$_3$](ONf)$_2$ (**2e**)	-1.71	5.5	9.2	11.2	12.4

[a]V vs $Fc^{0/+}$ in MeCN.[11-13] [b]Yield based on $acrH_2$.

Supplementary Table 4. Catalytic ammonia formation using visible light: molybdenum catalysts.

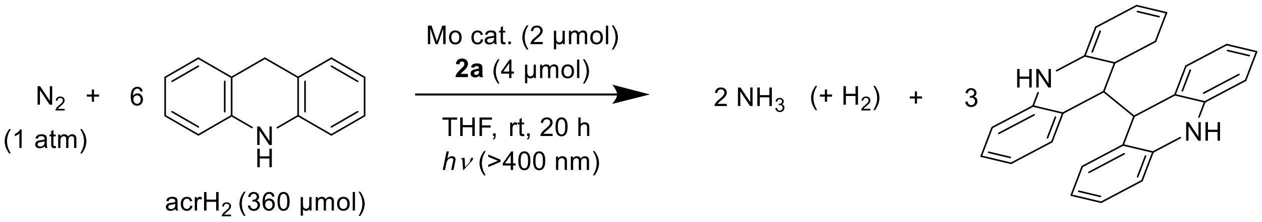

Mo cat.

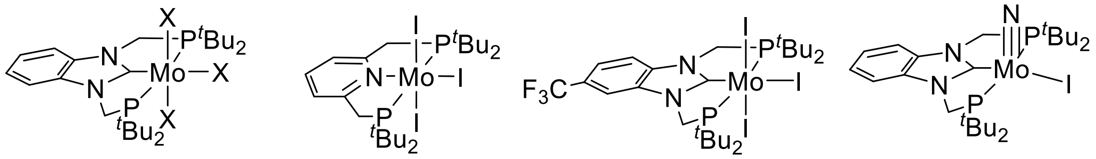

1a: X = I, **1b**: X = Cl   **1c**   **1d**   **1e**

Entry	Mo cat.	NH_3 (equiv/Mo)	NH_3 (%)[a]	H_2 (equiv/Mo)	H_2 (%)[a]
1	[MoI_3(PCP)] (**1a**)	29.5 ± 1.3	49.1 ± 1.3	33.7 ± 0.3	37.5 ± 0.4
2	[$MoCl_3$(PCP)] (**1b**)	0.5	0.8	5.7	6.3
3	[MoI_3(PNP)] (**1c**)	3.0	5.0	61.7	68.6
4	[MoI_3(CF_3-PCP)] (**1d**)	39.8 ± 3.1	66.2 ± 5.2	21.3 ± 7.2	23.6 ± 8.0
5[b]	[MoI_3(CF_3-PCP)] (**1d**)	41.3 ± 6.2	34.4 ± 5.2	12.0 ± 1.7	6.7 ± 0.9
6	[Mo(N)I(PCP)] (**1e**)	15.2	25.3	8.8	9.8

[a]Yield based on $acrH_2$. [b]$acrH_2$ (720 μmol) was used.

Isolation of (acrH)₂. A mixture of **1a** (1.8 mg, 0.0020 mmol), 9,10-dihydroacridine (65.2 mg, 0.36 mmol), and **2a** (4.3 mg, 0.0040 mmol) in THF (6 mL) was irradiated (> 400 nm) with stirring at room temperature for 20 h under N_2. The resultant white precipitate was collected by filtration, washed with THF, and dried in vacuo (48.0 mg, 0.13 mmol, 74%). Spectroscopic data were compared with previously reported literature.[14] Crystals suitable for X-ray analysis were prepared by recrystallization from DMSO–EtOH. Separately, we have confirmed after the reaction that 9,10-dihydroacridine was fully converted and that no formation of acridine was observed by ^{1}H NMR.

Catalytic ammonia formation under $^{15}N_2$. In a 50 mL Schlenk flask were placed **1a** (1.8 mg, 0.0020 mmol), 9,10-dihydroacridine (65.0 mg, 0.36 mmol), and **2a** (4.4 mg, 0.0040 mmol). The Schlenk flask was evacuated and then filled with $^{15}N_2$. THF (6 mL, stored under Ar) was added to the flask, and the mixture was irradiated (> 400 nm) with stirring at room temperature for 20 h. A solution of KO^{t}Bu (450 mg, 4.0 mmol) in THF (2.5 mL)-MeOH (2.5 mL) was added to the reaction mixture, and the mixture was stirred at room temperature for 15 min. The volatile components in the mixture were collected by trap-to-trap distillation, to which was added a solution of 1 M HCl in Et_2O

(2 mL, 2 mmol). Then, the mixture was stirred at room temperature. The resultant colorless solution was dried in vacuo to afford a white solid. The amount of formed $^{15}NH_4Cl$ was determined by ^1H NMR in DMSO-d_6 containing [ColH]OTf (8.3 mg, 0.031 mmol) as an internal standard (0.035 mmol, 18 equiv. based on the molybdenum atom, 29% NMR yield based on 9,10-dihydroacridine). ^1H NMR (DMSO-d_6): δ 7.23 (d, J = 71.2 Hz).

Synthesis of [Ir(ppy)$_2$(dtbbpy)] (3). To a solution of [Ir(ppy)$_2$(dtbbpy)]PF_6 (181 mg, 0.20 mmol) in THF (10 mL) was added a suspension of KC_8 (30 mg, 0.22 mmol) in THF (5 mL) at -78 °C, and the mixture was stirred at room temperature for 3 h. Volatiles were removed in vacuo, and toluene (3 mL) was added to the residue. The solution was filtered through Celite, and the filter cake was washed with toluene (1 mL × 7). After the extract was concentrated to about 5 mL, slow addition of hexane (15 mL) afforded **3** as brown crystals, which were collected by filtration, washed with hexane (1 mL × 3), and dried in vacuo (110 mg, 0.14 mmol, 72%). Anal. Calcd. for $C_{40}H_{40}IrN_4$: C, 62.48; H, 5.24; N, 7.29. Found: C, 62.92; H, 5.14; N, 7.11. Crystals (**3**·2 C_4H_8O) suitable for X-ray analysis were prepared by recrystallization from THF–hexane.

Reduction of 1a with 3 under N_2. To a solution of **1a** (9.1 mg, 0.010 mmol) in THF (1 mL) was added a solution of **3** (17.3 mg, 0.022 mmol) in THF (1 mL), and the mixture was stirred at room temperature for 30 min. The resultant yellow-brown suspension was filtered through Celite, and the filter cake was washed with THF (1 mL). Volatiles were removed in vacuo. The amount of [Mo(N)I(PCP)] (**1e**) was determined by ^1H NMR in DMSO-d_6 containing C_6Me_6 (9.0 mg, 0.056 mmol) as an internal standard (0.0088 mmol, 88% NMR yield). ^1H NMR (THF-d_8): δ 7.77 (dd, J = 3.2, 6.0 Hz, 2H), 7.39 (dd, J = 2.8, 6.8 Hz, 2H), 4.80 (d, J = 13.2 Hz, 2H), 4.67 (d, J = 13.6 Hz, 2H), 1.61 (pseudo t, 18H), 1.17 (pseudo t, 18H). $^{31}P\{^1H\}$ NMR (THF-d_8): δ 113.3 (s).

Stoichiometric photoreduction of 1e with 9,10-dihydroacridine under Ar. A mixture of **1e** (6.7 mg, 0.010 mmol), 9,10-dihydroacridine (7.2 mg, 0.040 mmol), and **2a** (4.3 mg, 0.0040 mmol) in THF (6 mL) was irradiated (> 400 nm) with stirring at room temperature for 20 h under Ar. The amount of ammonia (0.0094 mmol, 94% yield based on **1e**) was determined following general procedures.

Preparation of 9,9-dideuterio-9,10-dihydroacridine (dihydroacridine-*d*$_2$). To a solution of $NaBD_4$ (418 mg, 10 mmol) in diglyme (5 mL) was added a solution of I_2 (1298 mg, 5.1 mmol) in diglyme (5 mL) by dropping funnel over a period of 20 min with stirring. The gas evolved during the reaction was passed through a cooled solution of 9(10H)-acridone (977 mg, 5.0 mmol) in THF (20 mL) by cannula. After the addition of I_2, the THF solution of 9(10H)-acridone was stirred at room temperature for 20 min and then refluxed for 13 h. To the resultant solution were added brine (20 mL) and aqueous solution of NaOH (2 M, 5 mL), and the mixture was stirred. The organic phase

was collected, and the aqueous phase was extracted by Et_2O (5 mL x 3). The solvent was removed in vacuo, and then the crude mixture was purified by column chromatography (SiO_2) with hexane/AcOEt (90/10 to 70/30) to give dihydroacridine-d_2 as a colorless crystalline solid (807 mg, 4.4 mmol, 88 %). ^{1}H NMR ($CDCl_3$): δ 7.11-7.06 (m, 4H), 6.85 (dt, J = 0.8, 7.2 Hz, 2H), 6.67 (dd, J = 0.8, 7.6 Hz, 2H), 5.95 (s, 2H).

Kinetic study of catalytic reactions. Kinetic isotope effect (KIE) of the catalytic reactions was estimated by measuring the amount of ammonia for the catalytic reaction using dihydroacridine-d_2 as a proton source. A typical procedure is described below. A mixture of **1a** (1.8 mg, 0.0020 mmol), dihydroacridine-d_2 (66.1 mg, 0.36 mmol), and **2a** (4.4 mg, 0.0040 mmol) in THF (6 mL) was irradiated (> 400 nm) with stirring at room temperature for 2 h under N_2. The amount of ammonia (0.0071 mmol, 6% yield based on dihydroacridine-d_2) was determined following general procedures. The results of the catalytic reactions were summarized in **Supplementary Table 5**.

Supplementary Table 5. Catalytic ammonia formation using visible light (for 2 h)

	ammonia (mmol)	Yield (%)[a]
dihydroacridine	0.0213	18
dihydroacridine-d_2	0.0071	6
		KIE = 3.0

[a]Yield based on dihydroacridine (dihydroacridine-d_2).

1.3 Cyclic voltammetric studies

Cyclic voltammograms were recorded on a potentiostat/galvanostat (GAMRY INSTRUMENTS, Interface 1000) with glassy carbon disk (diameter 3 mm) as a working electrode and Pt wire as a counter electrode in THF or MeCN containing 1 mM of sample and 0.1 M of [^{n}Bu$_4$N][PF$_6$] as a supporting electrolyte at a scan rate of 0.1 V/s at room temperature under N_2 atmosphere. All potentials were measured against an $Ag^{0/+}$ electrode and converted to the values *vs.* ferrocene/ferrocenium ($FeCp_2^{+/0}$) referenced to external standard ($FeCp_2^{+/0}$). Cyclic voltammograms of [Ir(ppy)$_2$(dtbbpy)]ONf (**2a**), acrH_2, [Ir(ppy)$_2$(dtbbpy)] (**3**), [MoI$_3$(PCP)] (**1a**), and [Mo(N)I(PCP)] (**1e**) are shown in **Supplementary Figures 1-6**, respectively.

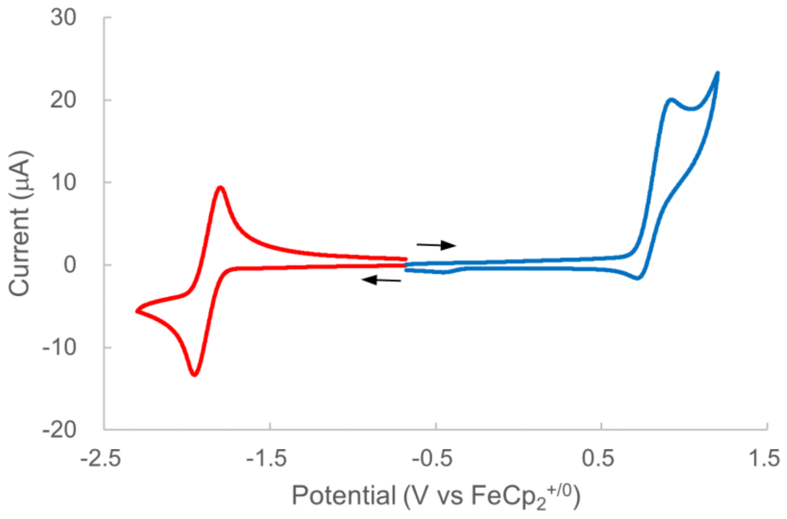

Supplementary Figure 1. Cyclic voltammograms of **2a** in THF. $E_{1/2(red)}$ = -1.88 V (vs $FeCp_2^{+/0}$).

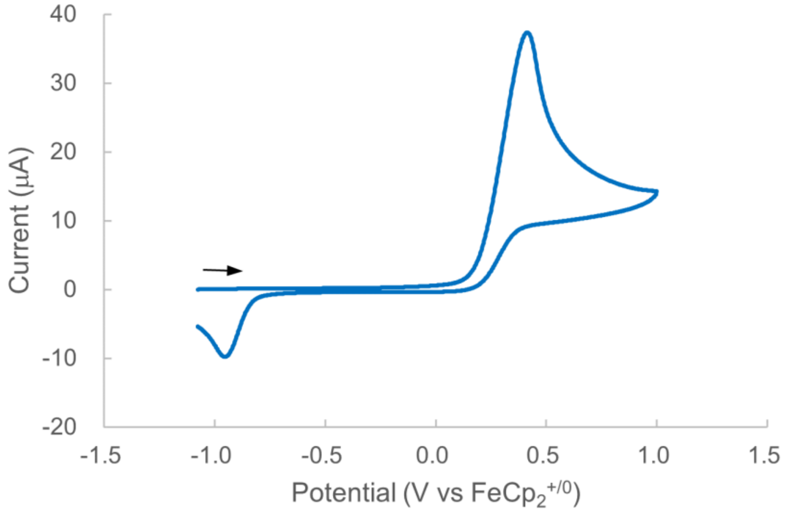

Supplementary Figure 2. Cyclic voltammograms of acrH_2 in THF. E_{pa} = +0.41 V (vs $FeCp_2^{+/0}$).

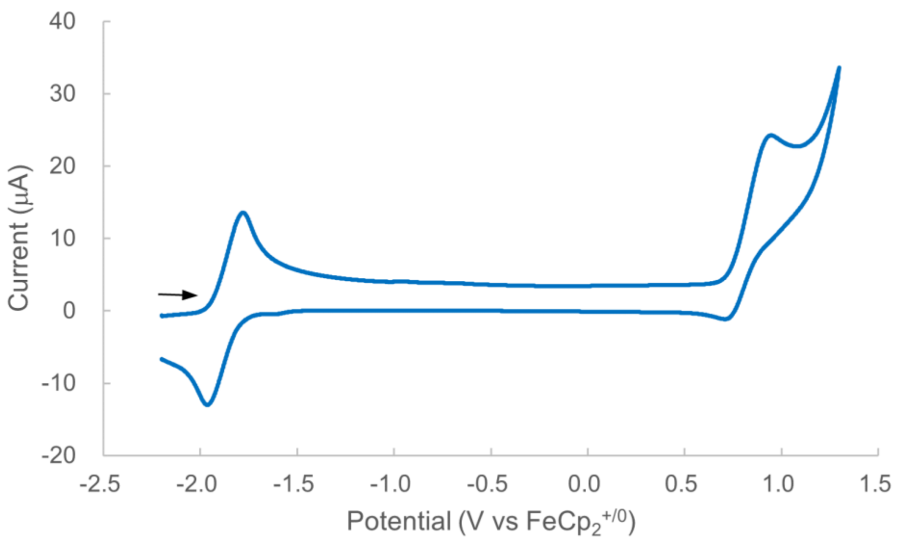

Supplementary Figure 3. Cyclic voltammograms of **3** in THF. $E_{1/2(ox)}$ = -1.87 V (vs $FeCp_2^{+/0}$).

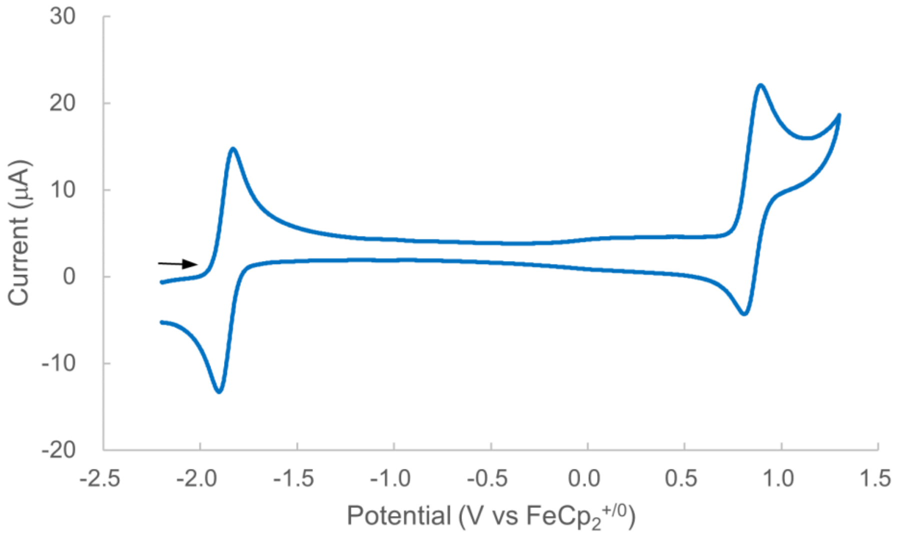

Supplementary Figure 4. Cyclic voltammograms of **3** in MeCN. $E_{1/2(ox)}$ = -1.87, +0.85 V (vs $FeCp_2^{+/0}$).

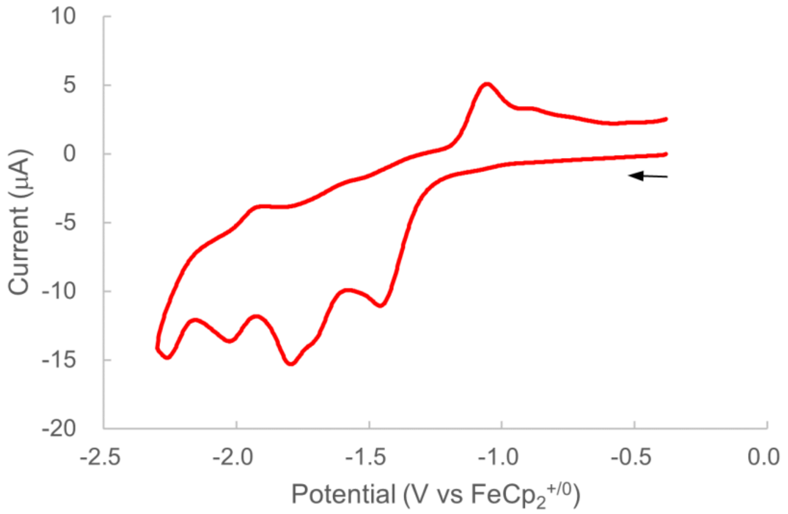

Supplementary Figure 5. Cyclic voltammograms of **1a** in THF. E_{pc} = -1.46, -1.80, -2.03 V (vs $FeCp_2^{+/0}$).

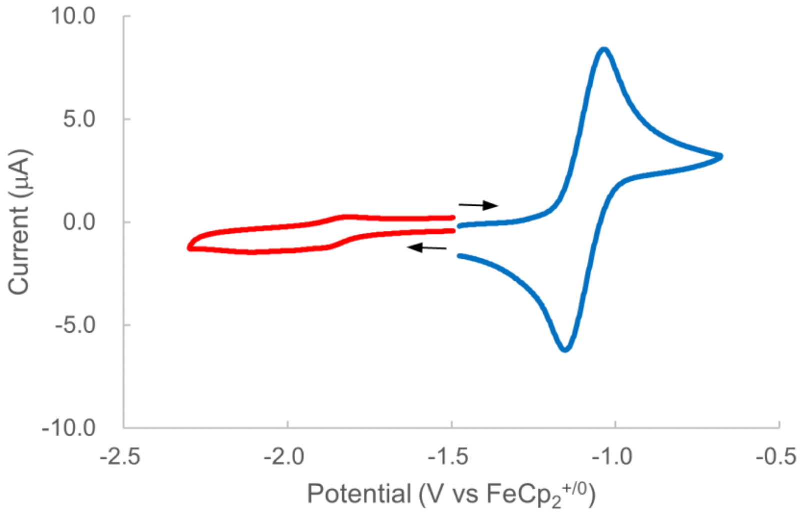

Supplementary Figure 6. Cyclic voltammograms of **1e** in THF. $E_{1/2(ox)}$ = -1.10 V (vs $FeCp_2^{+/0}$).

1.4 NMR spectra.

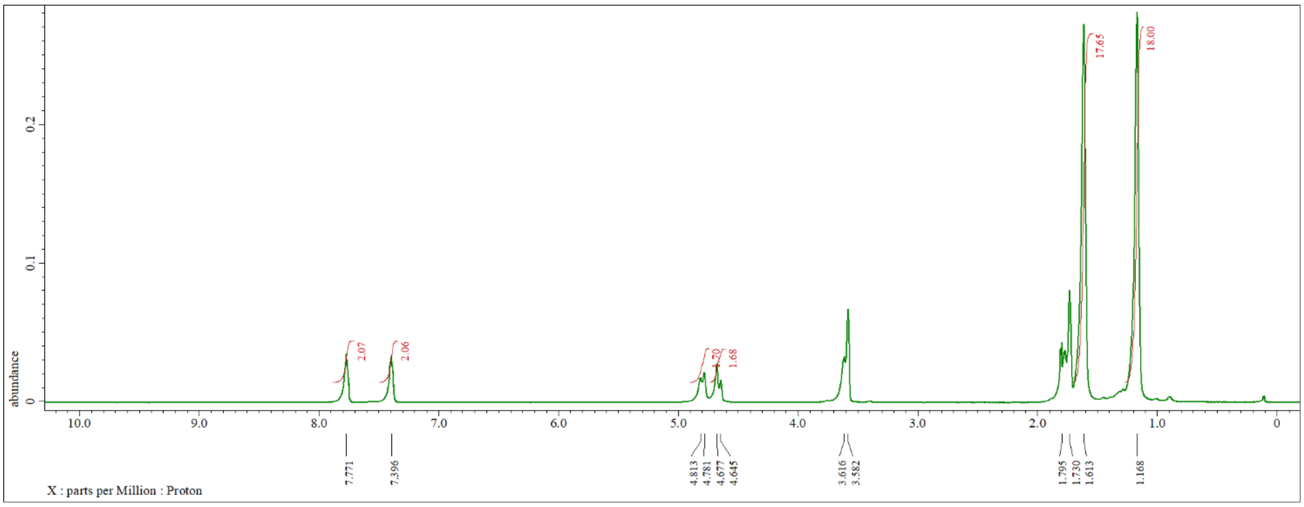

Supplementary Figure 7. ^{1}H NMR spectrum of [Mo(N)I(PCP)] (**1e**) in THF-d_8.

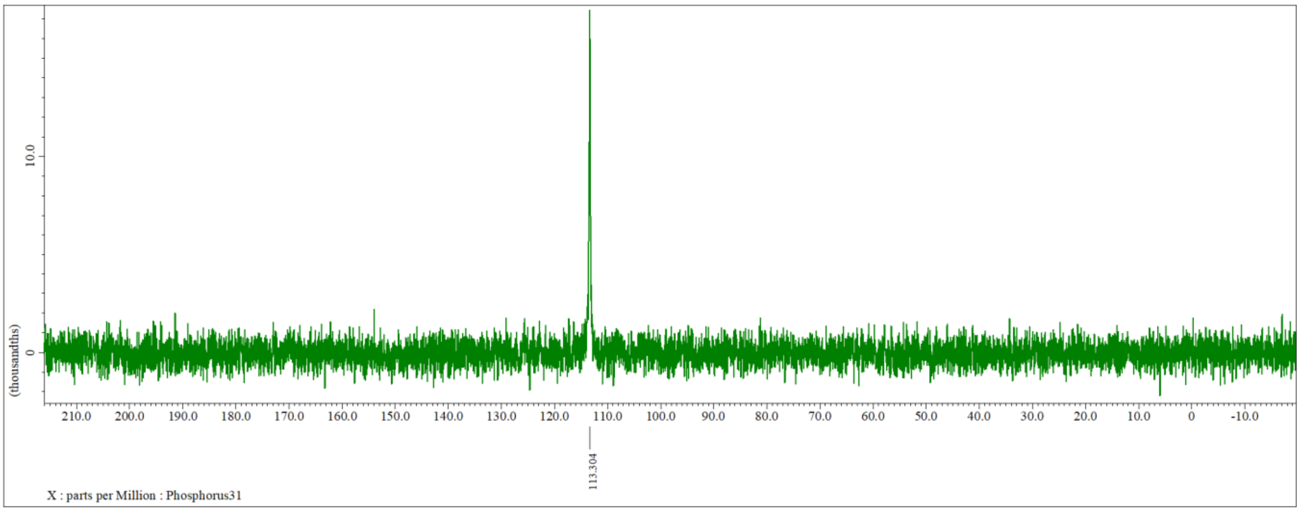

Supplementary Figure 8. $^{31}P\{^{1}H\}$ NMR spectrum of [Mo(N)I(PCP)] (**1e**) in THF-d_8.

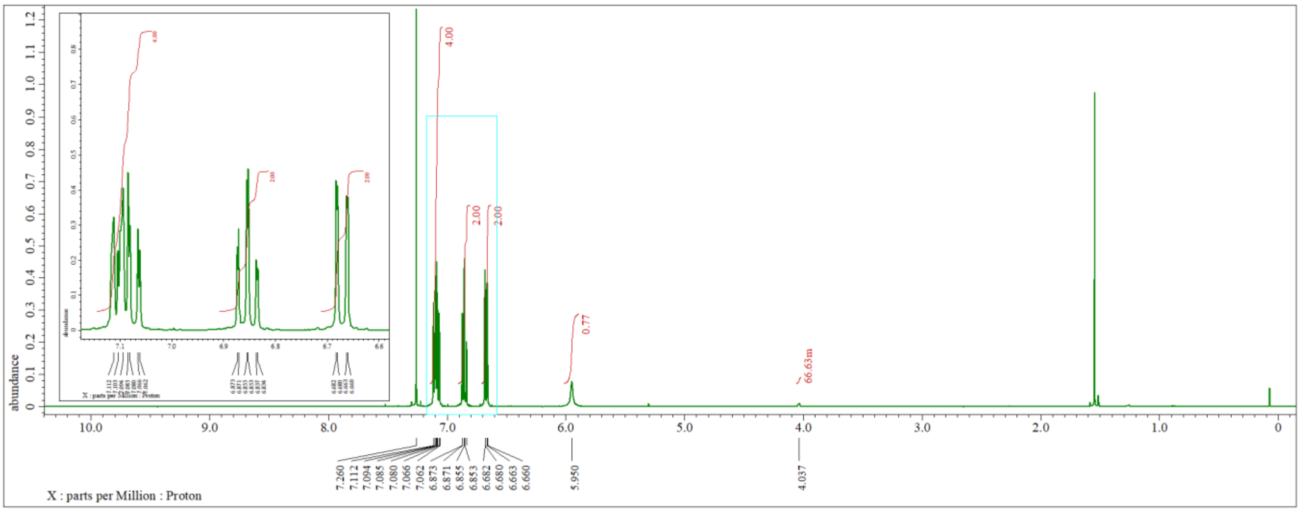

Supplementary Figure 9. ^{1}H NMR spectrum of dihydroacridine-d_2 in $CDCl_3$

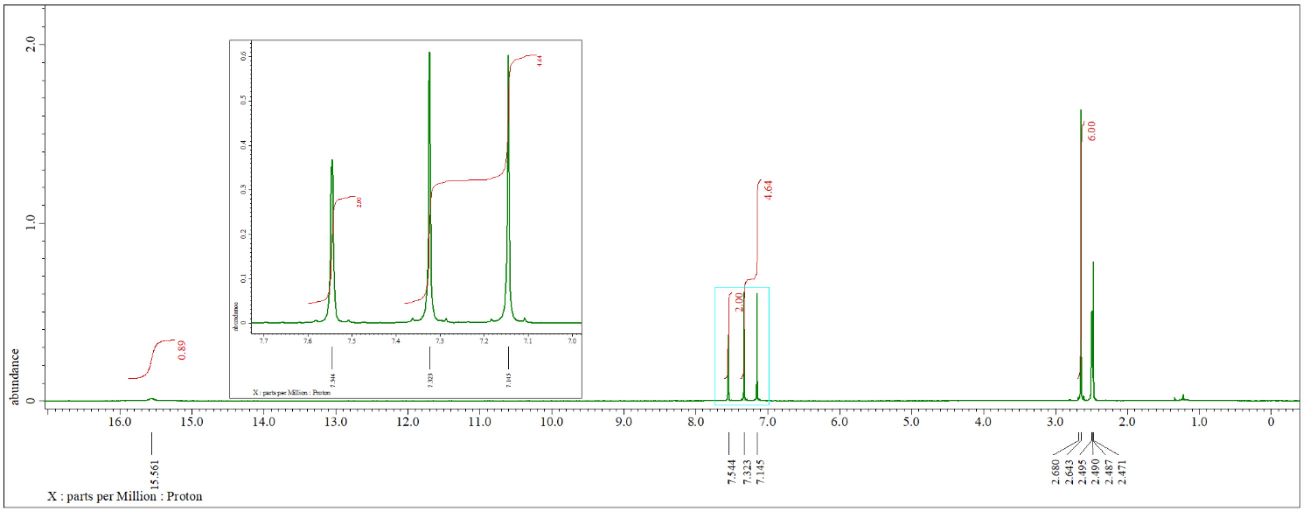

Supplementary Figure 10. ^{1}H NMR spectrum after the catalytic ammonia formation under $^{15}N_2$ in DMSO-d_6 containing [ColH]OTf as an internal standard.

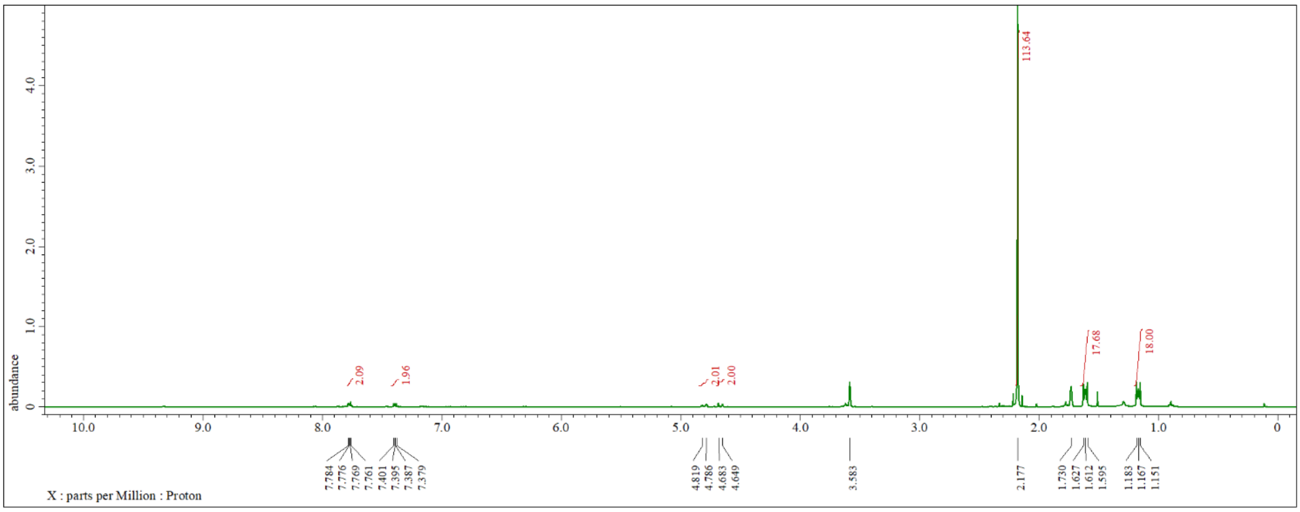

Supplementary Figure 11. ^{1}H NMR spectrum of the reaction of [MoI_3(PCP)] (**1a**) with [Ir(ppy)$_2$(dtbbpy)] (**3**) under N_2 in THF-d_8 containing C_6Me_6 as an internal standard.

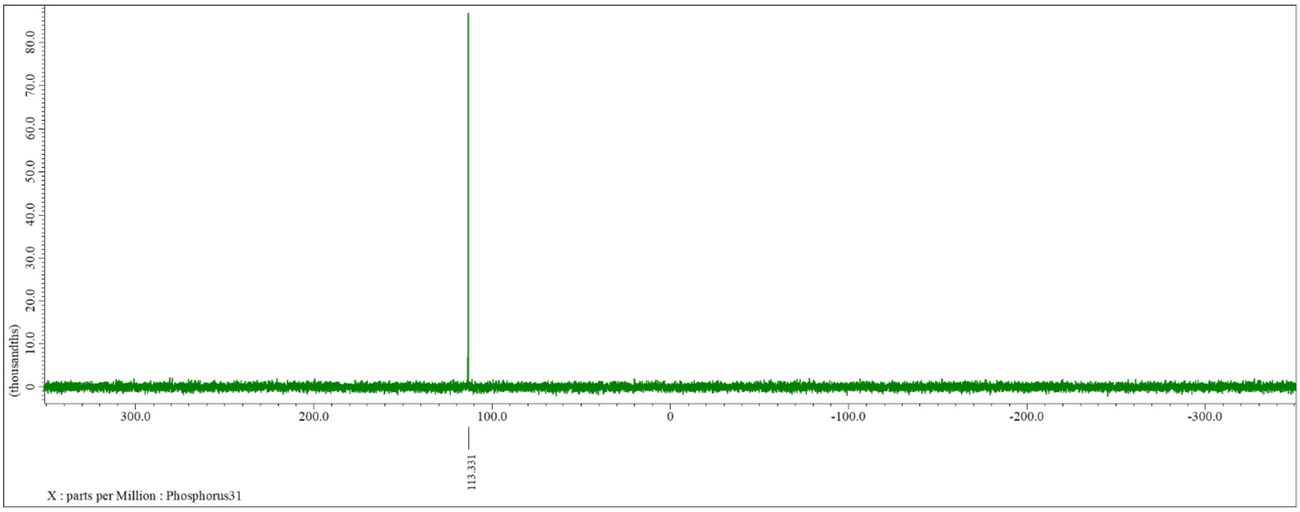

Supplementary Figure 12. $^{31}P\{^{1}H\}$ NMR spectrum of the reaction of [MoI_3(PCP)] (**1a**) with [Ir(ppy)$_2$(dtbbpy)] (**3**) under N_2 in THF-d_8.

1.5 UV-vis spectra

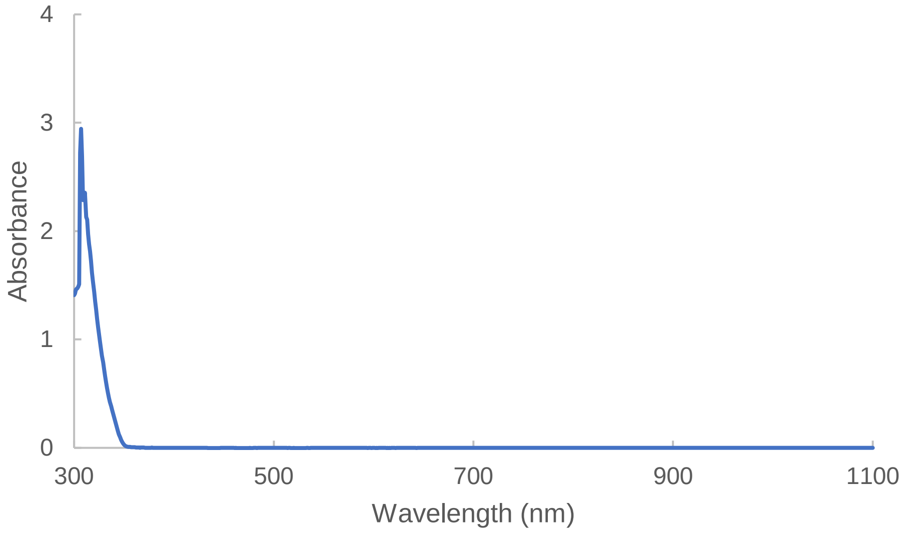

Supplementary Figure 13. UV-vis spectrum of acrH_2 in THF (3.0 x 10^{-4} M).

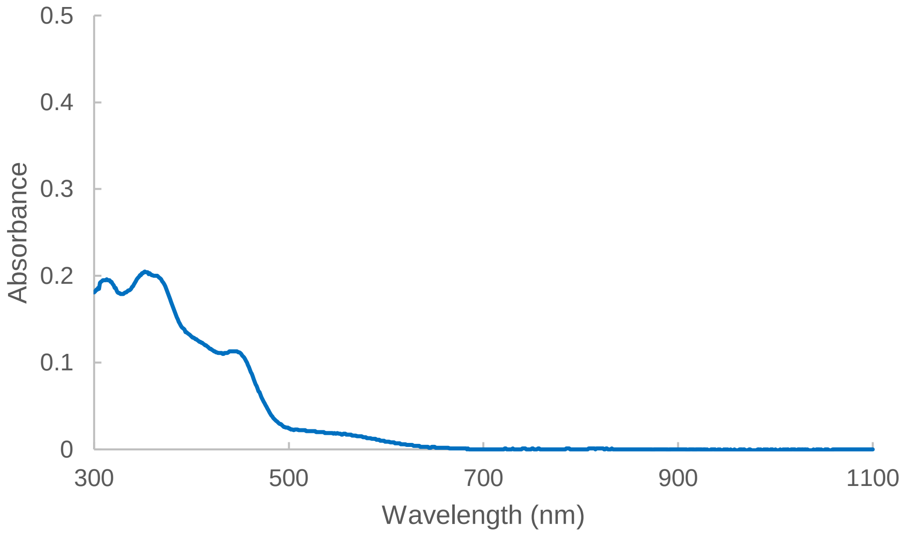

Supplementary Figure 14. UV-vis spectrum of **1a** in THF (2.0 x 10^{-5} M).

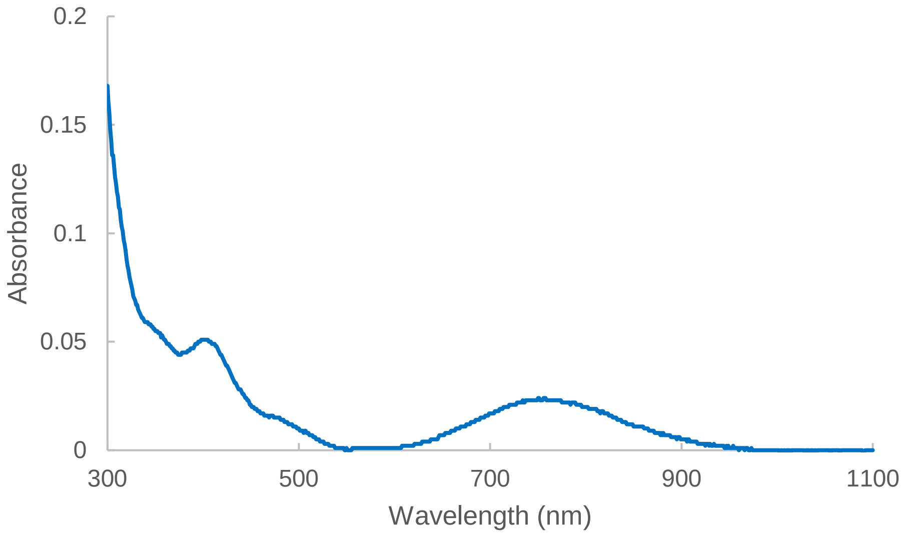

Supplementary Figure 15. UV-vis spectrum of **1e** in THF (2.0 x 10^{-5} M).

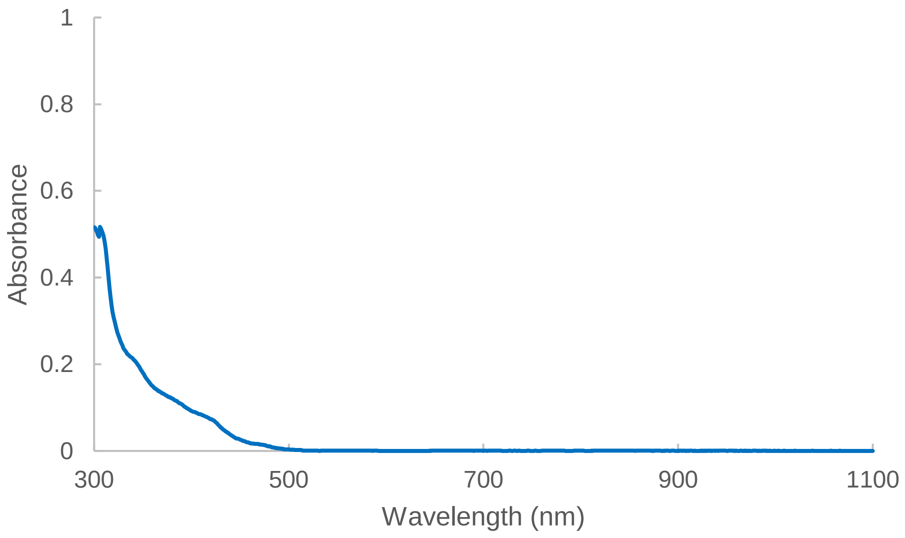

Supplementary Figure 16. UV-vis spectrum of **2a** in THF (2.0 x 10^{-5} M).

1.6 Light ON/OFF experiments

In a 50 mL Schlenk flask were placed **1a** (0.0020 mmol), 9,10-dihydroacridine (0.36 mmol), and **2a** (4.4 mg, 0.0040 mmol). The Schlenk flask was evacuated and then filled with N_2. THF (6 mL) was added to the flask, and the mixture was stirred at room temperature under irradiation (> 400 nm) or dark (Schlenk flask was wrapped with aluminum foil) for indicated time. The amount of ammonia was determined following general procedures. The results are shown in **Supplementary Table 6**.

Supplementary Table 6. Light ON/OFF experiments for catalytic ammonia formation.

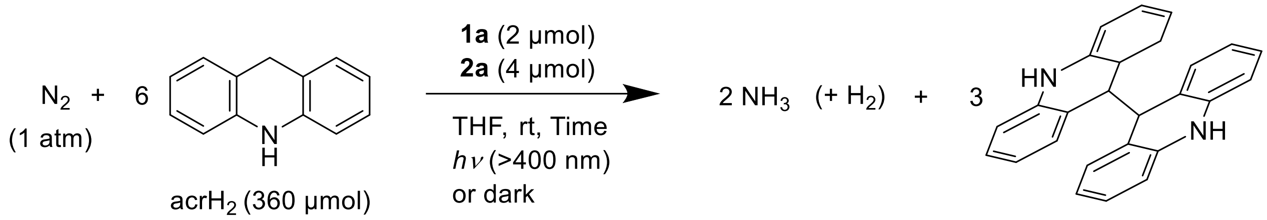

Entry	Time (h)	Photo irradiation	NH_3 (equiv/Mo)	NH_3 (%)[a]
1	2	ON (2 h)	10.7	18
2	3	ON (2 h)→OFF (1 h)	10.8	18
3	4	ON (2 h)→OFF (1 h)→ON (1 h)	22.2	37
4	5	ON (2 h)→OFF (1 h)→ON (1 h)→OFF (1 h)	21.7	36
5	6	ON (2 h)→OFF (1 h)→ON (1 h)→OFF (1 h)→ON (1 h)	26.8	44

[a]Yield based on $acrH_2$.

1.7 Stern-Volmer analysis

Stern-Volmer luminescence quenching studies for the THF solution of [Ir(ppy)$_2$(dtbbpy)]ONf (**2a**, 4.0×10^{-6} M) with variable concentrations of 9,10-dihydroacridine (acrH_2) were performed on a Shimadzu RF-5300PC spectrophotometer under N_2 atmosphere. The solutions were irradiated at 420 nm, and the luminescence was measured at 570 nm. The results are shown in **Supplementary Figure 17** and **Supplementary Table 7**. The Stern-Volmer plot (K_{SV} = 2291 M^{-1}) indicates that acrH_2 quenches the exited state of **2a** (**2a***).

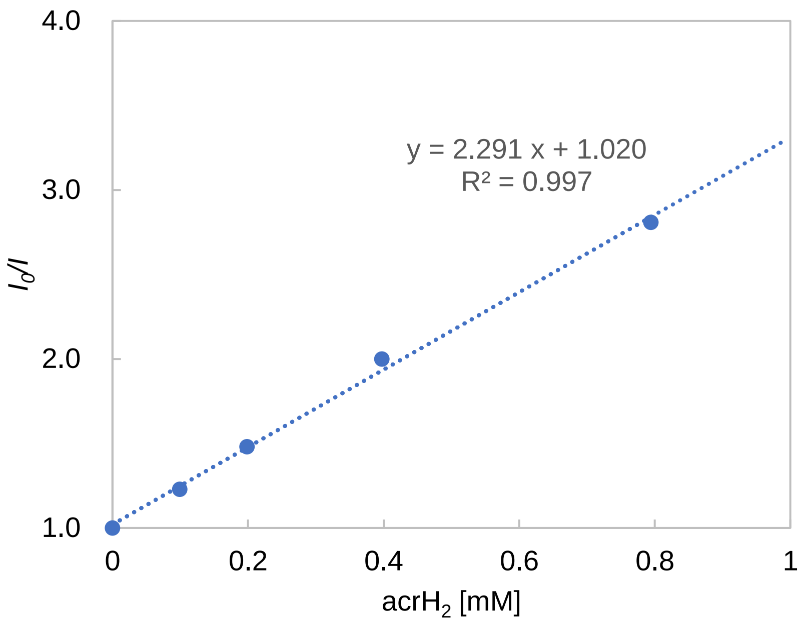

Supplementary Figure 17. Stern-Volmer luminescence quenching studies of **2a** with acrH_2.

Supplementary Table 7. Emission intensity of photocatalyst with variable concentrations of acrH_2.

acrH_2 (M)	0	0.99 × 10^{-4}	2.0 × 10^{-4}	4.0 × 10^{-4}	8.0 × 10^{-4}
I	274	223	185	137	97.7
I_0/I	1	1.23	1.48	2.00	2.81

1.8 Determination of quantum yield

In a 50 mL Schlenk flask were placed **1a** (1.8 mg, 0.0020 mmol), 9,10-dihydroacridine (65.2 mg, 0.36 mmol), and **2a** (4.3 mg, 0.0040 mmol). The Schlenk flask was evacuated and then filled with N_2. THF (6 mL) was added to the flask, and the mixture was irradiated using mercury lamp USH 250SC2 (250 W) equipped with a 410 nm band pass filter (Kenko B410) for 2 h. The amount of ammonia (0.0403 mmol, 20.2 equiv/Mo, 33.6% yield based on 9,10-dihydroacridine) was determined following general procedures. The irradiated light intensity to a 6.0 mL solution in 50 mL Schlenk flask was estimated to be 8.50×10^{-7} E s^{-1} at 410 nm bandpass filter (Kenko B410) by using $K_3[Fe(C_2O_4)_3]$ as a chemical actinometer.[15] Thus, the quantum yield of the catalytic reaction by **1a** and **2a** is given as $\Phi = 6.6 \times 10^{-3}$ using the following equation:

$$\Phi = \frac{n_{product}}{flux \cdot t} = \frac{40.3 \times 10^{-6}}{8.5 \times 10^{-7} \times 7200} = 6.6 \times 10^{-3}$$

1.9 X-Ray crystallographic studies.

Diffraction data for $(acrH)_2$ and $[Ir(ppy)_2(dtbbpy)]\cdot 2\ C_4H_8O$ (**3**$\cdot 2\ C_4H_8O$) were collected for the 2θ range of 4° to 60° at – 180 °C on a Rigaku XtaLAB Synergy-S diffractometer equipped with a HyPix-6000HE Hybrid Photon Counting (HPC) detector and VariMax optics using multi-layer mirror monochromated Mo Kα radiation (λ = 0.71073 Å). Intensity data were corrected for Lorenz-polarization effects and for empirical absorption (CrysAlisPro),[16] whereas structure solutions and refinements were carried out by using the *CrystalStructure* crystallographic software package.[17] Positions of non-hydrogen atoms were determined by direct methods (SHELXT Version 2014/5 for $(acrH)_2$, SIR2011 for **3**$\cdot 2\ C_4H_8O$)[18,19] and subsequent Fourier syntheses (SHELXL Version 2016/6),[20] and were refined on F_o^2 using all unique reflections by full-matrix least-squares with anisotropic thermal parameters except for several disordered atoms summarized below, which were solved isotropically. All the other hydrogen atoms were placed at the calculated positions with fixed isotropic parameter except for several hydrogen atoms summarized below, which could not be placed at calculated positions mainly because of disorders. Anomalous dispersion effects were included in F_c,[21] and mass attenuation coefficients, values for $\Delta f'$ and $\Delta f''$, and neutral atom scattering factors were taken from references.[22–24] Details of the crystal and data collection parameters are summarized in **Supplementary Table 8**, and ORTEP drawings of molecular structures are shown in **Supplementary Figures 18** and **19**.

There are two B-level alerts for crystallographic data of **3**$\cdot 2\ C_4H_8O$ (PLAT971_ALERT_2_B Check Calcd Resid. Dens. 1.83 Ang from C23 3.37 eA^{-3}, PLAT971_ALERT_2_B Check Calcd Resid. Dens. 1.74 Ang From C32 3.17 eA^{-3}). These two residual peaks may be due to two additional possible disorders of molecules. These are due to several minor disorders of iridium atoms, although such minor disorders were not solved here.

Supplementary Table 8. X-Ray crystallographic data for $(acrH)_2$, **3**·2 C_4H_8O.

	$(acrH)_2$	**3**·2 C_4H_8O
chemical formula	$C_{26}H_{20}N_2$	$C_{48}H_{56}IrN_4O_2$
CCDC number	2189300	2189301
formula weight	360.46	913.22
dimensions of crystals,	0.259 × 0.065 × 0.049	0.298 × 0.104 × 0.070
crystal color, habit	colorless, block	brown, block
crystal system	monoclinic	triclinic
space group	$P2_1/n$	P-1
a, Å	12.5959(5)	13.8107(4)
b, Å	5.4750(2)	13.9698(6)
c, Å	12.6846(5)	14.7479(6)
α, deg	90	64.518(4)
β, deg	96.443(4)	82.412(3)
γ, deg	90	71.725(3)
V, Å^3	869.24(6)	2438.99(18)
Z	2	2
ρ_{calcd}, g cm^{-3}	1.377	1.243
F(000)	380.00	930.00
μ, cm^{-1}	0.806	27.817
temperature, °C	–180	–180
trans. factors range	0.628 – 0.996	0.556 – 0.823
no. reflections measured	6300	36675
no. unique reflections	2156 (R_{int} = 0.0259)	12014 (R_{int} = 0.0451)
no. parameters refined	131	496
$R1$ ($I > 2\sigma(I)$)[a]	0.0445	0.0386
$wR2$ (all data)[b]	0.1238	0.0861
GOF (all data)[c]	1.045	1.002
max diff peak / hole, e Å^{-}	+0.44 / –0.25	+3.46 / –1.85

[a] $R1 = \Sigma||F_o|-|F_c||/\Sigma|F_o|$. [b] $wR2 = [\Sigma w(F_o^2-F_c^2)^2/\Sigma w(F_o^2)^2]^{1/2}$, $w = 1/[\sigma^2(F_o^2) + (qP)^2 + rP]$, $P = (\mathrm{Max}(F_o^2, 0) + 2F_c^2)/3$ [q = 0.0672 ($(acrH)_2$), 0.0531 (**3**·2 C_4H_8O); r = 0.3941 ($(acrH)_2$), 0 (**3**·2 C_4H_8O)]. [c] GOF = $[\Sigma w(F_o^2-F_c^2)^2/(N_o-N_{params})]^{1/2}$.

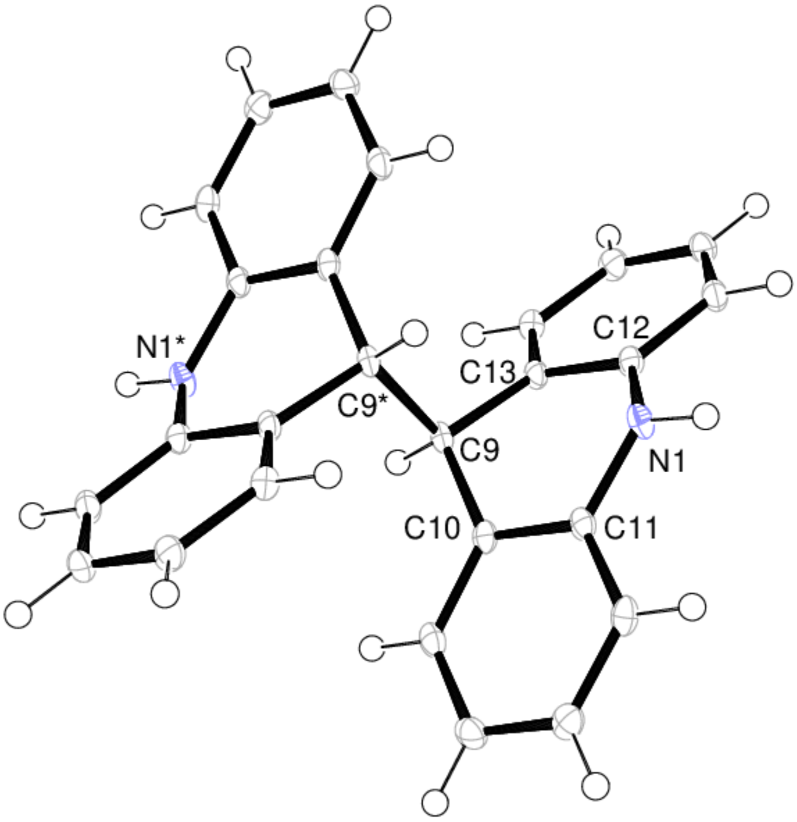

Supplementary Figure 18. Molecular structure of $(acrH)_2$. Thermal ellipsoids are shown at the 50% probability level. Selected bond lengths (Å) and angles (deg): N1–C11 1.4052(17), N1–C12 1.4034(17), C9–C10 1.5098(17), C10–C11 1.4071(17), C12–C13 1.4029(17), C9–C13 1.5108(18), C9–C9* 1.5888(16), C11–N1–C12 118.59(11), C10–C9–C13 109.16(9), C9–C10–C11 117.75(11), C10–C11–N1 118.49(11), N1–C12–C13 118.54(11), C9–C13–C12 117.91(11).

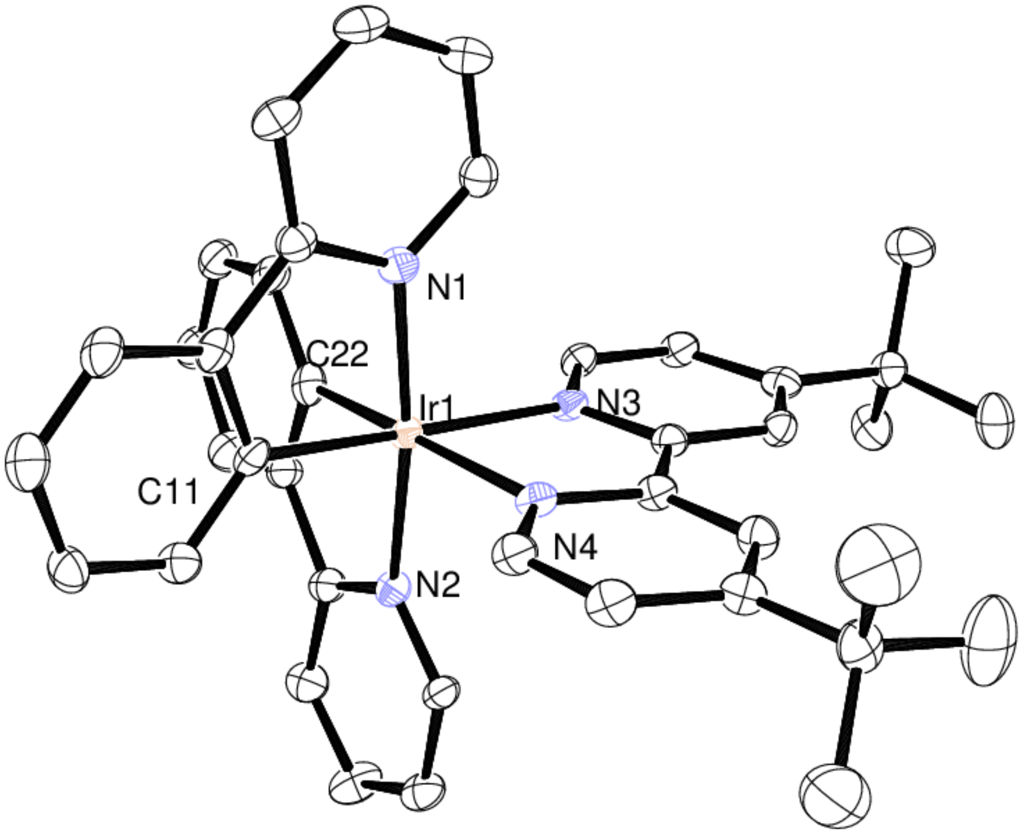

Supplementary Figure 19. Molecular structure of **3**. Thermal ellipsoids are shown at the 50% probability level, and hydrogen atoms are omitted for clarity. Selected bond lengths (Å) and angles (deg): Ir1–N1 2.045(3), Ir1–N2 2.035(3), Ir1–N3 2.124(3), Ir1–N4 2.130(3), Ir1–C11 2.026(4), Ir1–C22 2.015(4), N1–Ir1–C11 80.43(14), N2–Ir1–C22 80.50(15), N3–Ir1–N4 77.23(12).

1.10 Computational Details.

Density-functional-theory (DFT) calculations were performed with the Gaussian 09 program (Rev. E01).[25] All geometry optimizations were carried out with the B3LYP functional with the Grimme's dispersion correction (B3LYP-D3).[26-30] We employed the SDD (Stuttgart/Dresden pseudopotentials) basis set[31,32] for Mo, I and Ir and the 6-31G(d) basis set[33-36] for the other atoms, respectively. Optimized structures were confirmed to have no imaginary frequencies by vibrational analysis. **Supplementary Figures 20** and **21** presents optimized chemical species used for the discussion on possible reaction pathways for the transformation of the Mo-nitride complex to the Mo-imide complex. To discuss the energetics, single-point energy calculations were performed for all optimized structures using the SDD and the 6-311+G(d,p) basis sets.[37-39] In the single-point calculations, solvation effects of THF (ε = 7.4257) were taken into account by using the polarizable continuum model (PCM).[40] Calculated energy profiles of the possible reaction pathways are described in **Supplementary Figure 22**. Detailed data on SCF energies, thermal energy corrections at 298 K, and SCF energies in THF are summarized in **Supplementary Table 9**.

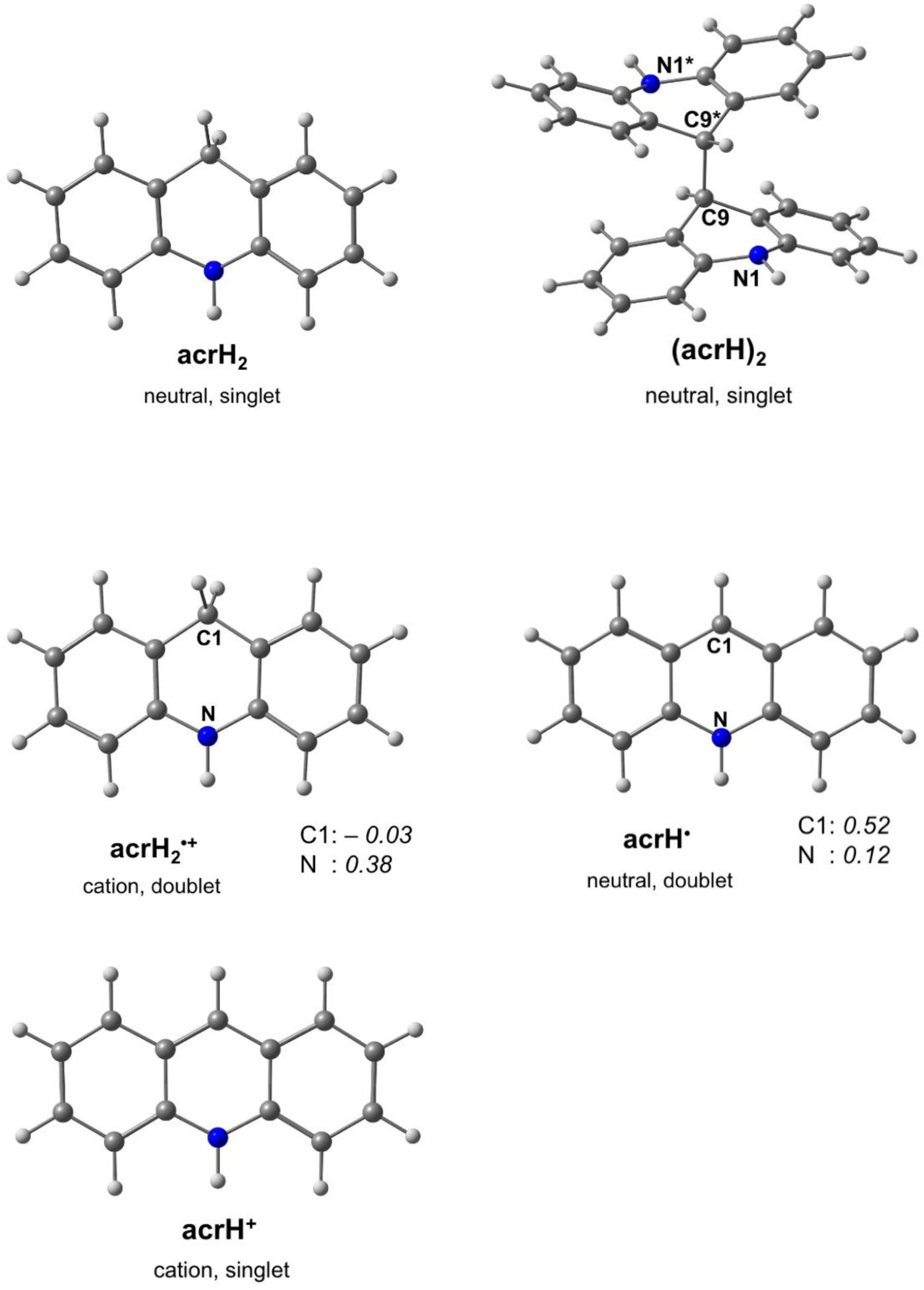

Supplementary Figure 20. Optimized structures of $acrH_2$ and $(acrH)_2$, $acrH_2^{\bullet +}$, $acrH_2^{\bullet}$ and $acrH^+$ in their ground spin states. The Mulliken spin densities are given in italics.

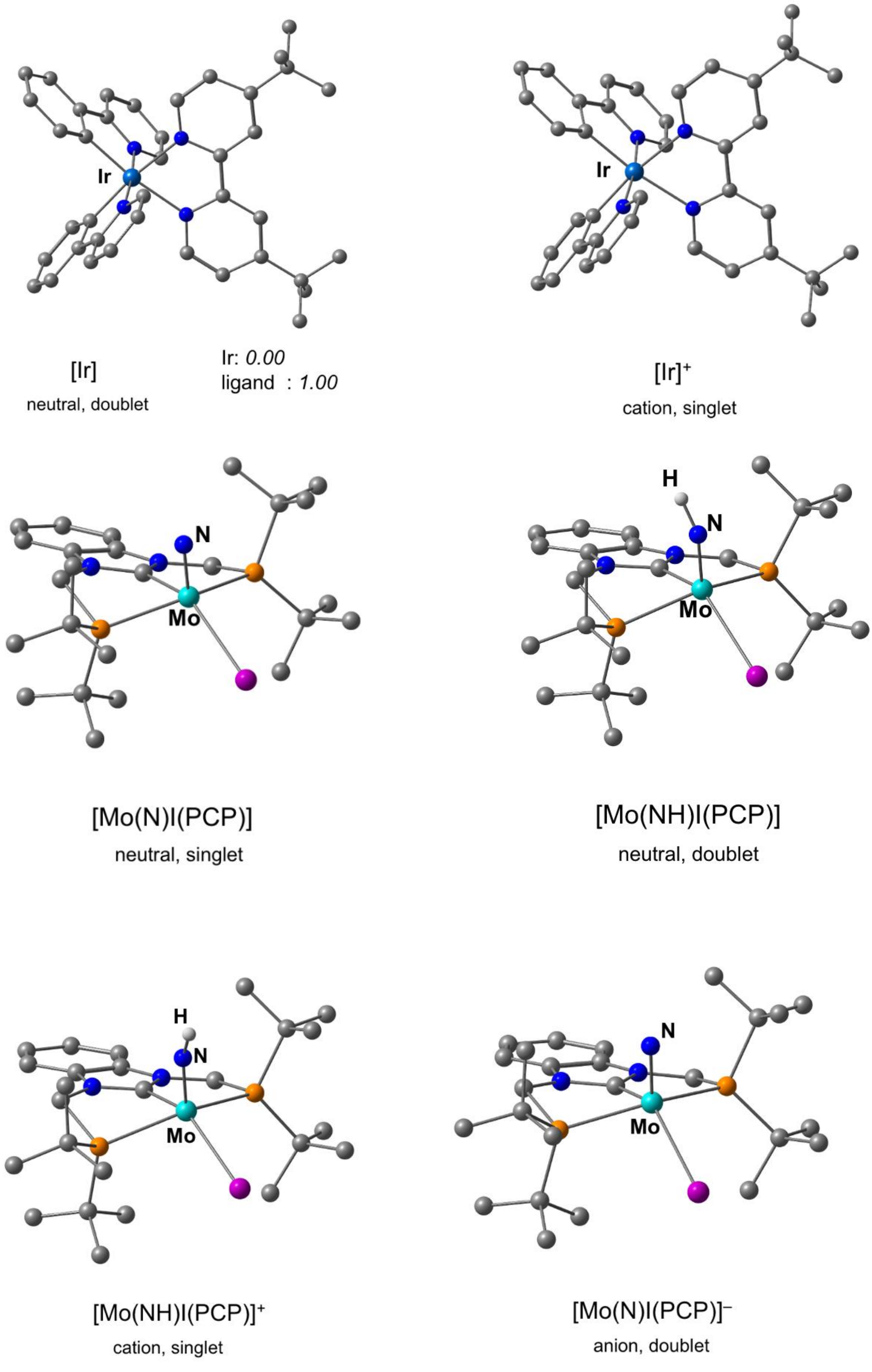

Supplementary Figure 21. Optimized structures of [Ir], [Ir]$^+$, Mo–nitride, and Mo–imide complexes in their ground spin states. Hydrogen atoms attached to carbons in Ir complexes and Mo complexes are omitted for clarity. The Mulliken spin densities are given in italics.

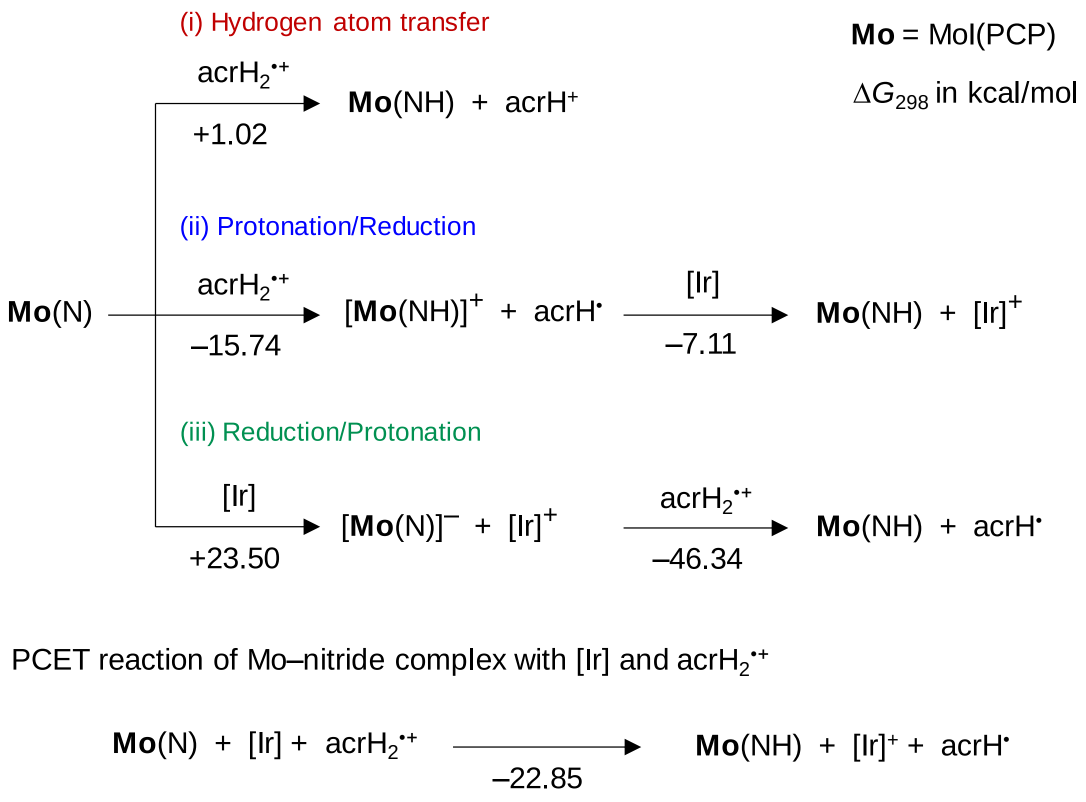

Supplementary Figure 22. Free energy profiles (ΔG_{298} in THF) of possible reaction pathways calculated for the transformation of [MoI(≡N)(PCP)] into [MoI(=NH)(PCP)].

Supplementary Table 9. SCF energies (*in vacuo*), thermal energy corrections at 298 K, SCF energies in THF.

Species	SCF energy /hartree	Thermal corrections /hartree	SCF energy (THF) /hartree
**acrH_2**	-556.79540221	0.170327	-556.94626652
**(acrH)$_2$**	-1112.40467152	0.343691	-1112.69783862
**acr$H_2^{\bullet+}$**	-556.55613311	0.169903	-556.75664143
**acr$H^{\bullet}$**	-556.17386215	0.157356	-556.32332730
**acrH^{+}**	-555.98252222	0.162013	-556.18140726
**[Ir]**	-1871.95567249	0.633881	-1872.44094835
**$[Ir]^{+}$**	-1871.82114234	0.638130	-1872.33664861
**[Mo(N)I(PCP)]**	-1905.97167182	0.593500	-1906.37626513
**[Mo(NH)I(PCP)]**	-1906.54540873	0.602862	-1906.95135225
**$[Mo(NH)I(PCP)]^{+}$**	-1906.38710363	0.605379	-1906.8339968
**$[Mo(N)I(PCP)]^{-}$**	-1905.98710519	0.587573	-1906.44144237

2. Supplementary references.

1. Eizawa, A., Arashiba, K., Egi, A., Tanaka, H., Nakajima, K., Yoshizawa, K. & Nishibayashi, Y. Catalytic reactivity of molybdenum-trihalide complexes bearing PCP-type pincer ligands. *Chem. Asian J.* **14**, 2091−2096 (2019).
2. Eizawa, A., Arashiba, K., Tanaka, H., Kuriyama, S., Matsuo, Y., Nakajima, K., Yoshizawa, K. & Nishibayashi, Y. Remarkable catalytic activity of dinitrogen-bridged dimolybdenum complexes bearing NHC-based PCP-pincer ligands toward nitrogen fixation. *Nat. Commun.* **8**, 14874 (2017).
3. Arashiba, K., Eizawa, A., Tanaka, H., Nakajima, K., Yoshizawa, K. & Nishibayashi, Y. Catalytic nitrogen fixation via direct cleavage of nitrogen–nitrogen triple bond of molecular dinitrogen under ambient reaction conditions. *Bull. Chem. Soc. Jpn.* **90**, 1111–1118, (2017).
4. Ashida, Y., Mizushima, T., Arashiba, K., Egi, A., Tanaka, H., Yoshizawa, K. & Nishibayashi, Y. Chemrxiv, (2022) doi:10.26434/chemrxiv-2022-jp6hz.
5. Miyake, Y., Ashida, Y., Nakajima, K. & Nishibayashi, Y. Visible-light-mediated addition of α-aminoalkyl radicals to [60]fullerene by using photoredox catalysts. *Chem. Eur. J.* **20**, 6120-6125 (2014).
6. Poli, R., Krueger, S. T. & Mattamana, S. P. Monomeric tetrahydrofuran-stabilized molybdenum(III) halaides. *Inorg. Synth*. **32**, 198-203 (1998).
7. Yandulov, D. V. & Schrock, R. R. Synthesis of tungsten complexes that contain hexaisopropylterphenyl-substituted triamidoamine ligands, and reactions relevant to the reduction of dinitrogen to ammonia. *Can. J. Chem*. **83**, 341-357 (2011).
8. Bain, G. A. & Berry, J. F. Diamagnetic corrections and Pascal's constants. *J. Chem. Educ*. **85**, 532-536 (2008).
9. Weatherburn, M. W. Phenol-hypochlorite reaction for determination of ammonia. *Anal. Chem.* **39**, 971–974 (1967).
10. Watt, G. W. & Chrisp, J. D. Spectrophotometric method for determination of hydrazine. *Anal. Chem.* **24**, 2006–2008 (1952).
11. Koike, T. & Akita, M. Visible-light radical reaction designed by Ru- and Ir-based photoredox catalysis. *Inorg. Chem. Front.* **1**, 562–576 (2014).
12. Garces, F. O., King, K. A. & Watts, R. J. Synthesis, structure, electrochemistry, and photophysics of methyl-substituted phenylpyridine ortho-metalated iridium(III) complexes. *Inorg. Chem.* **27**, 3464–3471 (1988).
13. Pavlishchuk, V. V. & Addison, A. W. Conversion constants for redox potentials measured versus different reference electrodes in acetonitrile solutions at 25°C. *Inorg. Chim. Acta.* **298**, 97–102 (2000).
14. Göth, H., Cerutti, P. & Schmid, H. Photoreaktionen von acridin und acridinabkömmlingen sowie von arklketonen mit methanol. *Helv. Chim. Acta* **48**, 1395-1406.
15. Hatchard C. G. & Parker, C. A. A new sensitive chemical actinometer-II. Potassium ferrioxalate

as a standard chemical actinometer. *Proc. R. Soc. London Ser. A* **235**, 518–526, (1956).
16. *CrysAlisPro*: Data Collection and processing software; Rigaku Corporation: Tokyo, Japan, 2015.
17. CrystalStructure 4.3.1: Single crystal structure analysis software; Rigaku Corp: Tokyo, Japan, and MSC: The Woodlands, TX, 2018.
18. Sheldrick, G. M. SHELXT – Integrated space-group and crystal-structure determination. *Acta Crystallogr. Sect. Found. Adv.* **71**, 3–8, (2015).
19. Burla, M. C., Caliandro, R., Camalli, M., Carrozzini, B., Cascarano, G. L., Giacovazzo, C., Mallamo, M., Mazzone, A., Polidori, G. & Spagna, R. SIR2011: a new package for crystal structure determination and refinement. *J. Appl. Crystallogr.* **45**, 357–361 (2012).
20. Sheldrick, G. M. Crystal structure refinement with SHELXL. *Acta Crystallogr. C* **71**, 3–8 (2015).
21. Ibers, J. A. & Hamilton, W. C. Dispersion corrections and crystal structure refinements. *Acta Crystallogr.* **17**, 781–782, (1964).
22. Creagh, D. C. & Hubbell, J.H. *International tables for crystallography*; Wilson, A. J. C. Ed.; Kluwer Academic Publishers: Dordrecht, 1992; vol. C,200.
23. Creagh, D. C. & McAuley, W. J. *International tables for crystallography*; Wilson, A. J. C. Ed.; Kluwer Academic Publishers: Dordrecht, 1992; vol. C, 219.
24. Maslen, E. N., Fox, A. G. & O'Keefe, M. A. *International tables for crystallography*; Wilson, A. J. C. Ed.; Kluwer Academic Publishers: Dordrecht, 1992; vol. C, 500.
25. *Gaussian 09, Revision E.01*: Frisch, M. J. et al. Gaussian, Inc.: Wallingford CT, 2013.
26. Becke, A. D. Density-functional exchange-energy approximation with correct asymptotic behavior. *Phys. Rev. A* **38**, 3098-3100 (1988).
27. Becke, A. D. Density-functional thermochemistry. III. The role of exact exchange. *J. Chem. Phys.* **98**, 5648-5652 (1993).
28. Lee, C., Yang, W. & Parr, R. G. Development of the Colle-Salvetti correlation-energy formula into a functional of the electron density. *Phys. Rev. B* **37**, 785-789 (1988).
29. Vosko, S. H., Wilk, L. & Nusair, M. J. Accurate spin-dependent electron liquid correlation energies for local spin density calculations: a critical analysis. *Can. J. Phys.* **58**, 1200-1211 (1980).
30. Grimme, S. Antony, J. Ehrlich, S. & Krieg, H. A consistent and accurate ab initio parametrization of density functional dispersion correction (DFT-D) for the 94 elements H-Pu. *J. Phys. Chem.* **132**, 154104 (2010).
31. Dolg, M., Wedig, U., Stoll, H. & Preuß, H. Energy-adjusted ab initio pseudopotentials for the first row transition elements. *J. Chem. Phys*. **86**, 866-872 (1987).
32. Andrae, D., Häußermann, U., Dolg, M., Stoll, H. & Preuß, H. Energy-adjustedab initio pseudopotentials for the second and third row transition elements. *Theor. Chim. Acta.* **77**, 123-141 (1990).

33. Ditchfield, R., Hehre, W. J. & Pople, J. A. Self-consistent molecular-orbital methods. IX. An extended Gaussian-type basis for molecular-orbital studies of organic molecules. *J. Chem. Phys*. **54**, 724-728 (1971).
34. Hehre, W. J., Ditchfield, R. & Pople, J. A. A. Self—consistent molecular orbital methods. XII. Further extensions of Gaussian—type basis sets for use in molecular orbital studies of organic molecules. *J. Chem. Phys.* **56**, 2257-2261 (1972).
35. Hariharan, P. C. & Pople, J. A. The influence of polarization functions on molecular orbital hydrogenation energies. *Theor. Chem. Acc.* **28**, 213-222 (1973).
36. Francl, M. M., Pietro, W. J., Hehre, W. J., Binkley, J. S., Gordon, M. S., DeFrees, D. J. & Pople, J. A. Self-consistent molecular orbital methods. XXIII. A polarization-type basis set for second-row elements. *J. Chem. Phys*. **77**, 3654-3665 (1982).
37. Krishnan, R., Binkley, J. S., Seeger, R. & Pople, J. A. Self-consistent molecular orbital methods. XX. A basis set for correlated wave functions. *J. Chem. Phys*. **72**, 650-654 (1980).
38. McLean, A. D. & Chandler, G. S. Contracted Gaussian basis sets for molecular calculations. I. Second row atoms, Z=11–18. *J. Chem. Phys*. **72**, 5639-5648 (1980).
39. Clark, T., Chandrasekhar, J., Spitznagel, G. W. & Schleyer, P. v. R. Efficient diffuse function-augmented basis sets for anion calculations. III. The 3-21+G basis set for first-row elements, Li–F. *J. Comput. Chem*. **4**, 294-301 (1983).
40. Tomasi, J., Mennucci, B. & Cammi, R. Quantum mechanical continuum solvation models. *Chem. Rev*. **105**, 2999-3094 (2005).
